# Longitudinal maturation of resting state networks: Relevance to sustained attention and attention deficit/hyperactivity disorder

**DOI:** 10.3758/s13415-022-01017-9

**Published:** 2022-06-08

**Authors:** Phoebe Thomson, Charles B. Malpas, Nandita Vijayakumar, Katherine A. Johnson, Vicki Anderson, Daryl Efron, Philip Hazell, Timothy J. Silk

**Affiliations:** 1 Department of Paediatrics, The University of Melbourne, Melbourne, Australia; 2 Murdoch Children’s Research Institute, Melbourne, Australia; 3 Melbourne School of Psychological Sciences, The University of Melbourne, Melbourne, Australia; 4 Department of Medicine, Royal Melbourne Hospital, The University of Melbourne, Melbourne, Australia; 5 School of Psychology, Deakin University, Melbourne, Australia; 6 The Royal Children’s Hospital, Melbourne, Australia; 7 Discipline of Psychiatry, The University of Sydney, Sydney, Australia

**Keywords:** Development, Attention deficit/hyperactivity disorder, Resting state fMRI, Functional connectivity, Attention, Longitudinal

## Abstract

The transition from childhood to adolescence involves important neural function, cognition, and behavior changes. However, the links between maturing brain function and sustained attention over this period could be better understood. This study examined typical changes in network functional connectivity over childhood to adolescence, developmental differences in attention deficit/hyperactivity disorder (ADHD), and how functional connectivity might underpin variability in sustained attention development in a longitudinal sample. A total of 398 resting state scans were collected from 173 children and adolescents (88 ADHD, 85 control) at up to three timepoints across ages 9-14 years. The effects of age, sex, and diagnostic group on changes in network functional connectivity were assessed, followed by relationships between functional connectivity and sustained attention development using linear mixed effects modelling. The ADHD group displayed greater decreases in functional connectivity between salience and visual networks compared with controls. Lower childhood functional connectivity between the frontoparietal and several brain networks was associated with more rapid sustained attention development, whereas frontoparietal to dorsal attention network connectivity related to attention trajectories in children with ADHD alone. Brain network segregation may increase into adolescence as predicted by key developmental theories; however, participants with ADHD demonstrated altered developmental trajectories between salience and visual networks. The segregation of the frontoparietal network from other brain networks may be a mechanism supporting sustained attention development. Frontoparietal to dorsal attention connectivity can be a focus for further work in ADHD.

**Supplementary Information:**

The online version contains supplementary material available at 10.3758/s13415-022-01017-9.

## Introduction

Childhood and adolescence are times of both significant functional brain development and cognitive change (Di Martino et al., 2014). Little is known, however, about within-individual functional connectivity changes that occur during development and how these changes might give rise to maturation of cognitive functions. This work uses a longitudinal design to map the maturation of network functional connectivity and their contribution to development of sustained attention over late childhood to early adolescence. It also considers the clinical relevance of these associations in the context of individuals with attention deficit/hyperactivity disorder (ADHD).

### Longitudinal Changes in Intrinsic Functional Connectivity over Development

Cross-sectional studies examining the development of functional connectivity have been reviewed thoroughly (Cao et al., 2016; Ernst et al., 2015; Grayson & Fair, 2017; Stevens, 2016) and generally support the principle that from childhood to early adolescence there is a decrease in between-network functional connectivity and increase in within-network functional connectivity. However, longitudinal work is necessary to tease apart potential inter- and intra-individual differences in these findings.

Existing longitudinal studies in childhood and late adolescence support the principle of increasing within-network and decreasing between-network connectivity across brain development (2-10 years: Long et al., 2017; Xiao et al., 2016; 13-22 years: Bernard et al., 2016; Strikwerda-Brown et al., 2015; Teeuw et al., 2019). Accelerated cohort studies suggest, however, that there may be critical periods such as the transition from childhood to adolescence in which greater changes in functional connectivity occur (Heyn et al., 2019; Jalbrzikowski et al., 2017; Peters et al., 2017; Wendelken et al., 2017). The transition to adolescence is a critical time for the emergence of psychopathology (Costello et al., 2011). Longitudinal studies assessing within-individual brain network development over this period are crucial for understanding neural trajectories related to these psychopathologies. Current studies have primarily focused on two networks (default mode and frontoparietal networks). Sherman et al. (2014) [scan ages 10 and 13, *N* = 45] observed increased functional connectivity within the default mode and frontoparietal networks, and decreased functional connectivity between the default mode and frontoparietal networks. Sylvester et al. (2018) with a larger sample [scan ages 10.5, 11.7, and 12.9, *N* = 147] failed to identify developmental changes in within default mode and frontoparietal network connectivity. Noting differences in these findings and consideration of only higher-order networks in Sherman et al. (2014) and Sylvester et al. (2018), the current work extends this literature by investigating a spectrum of networks along the functional hierarchy to clarify the pattern of network connectivity development over the late childhood to early adolescent period, as well as using longitudinal data to examine this process.

### Functional Connectivity Associated with Sustained Attention

Childhood and adolescence are periods of major advances in cognition, which are underpinned by neural development (Klausmeier & Allen, 2014; Stiles et al., 2015). Sustained attention is a cognitive domain which improves with age (Fortenbaugh et al., 2015) enabling increasing efficiency in avoiding internal and external distractions and maintaining attention (Ko et al., 2017). Several regions are implicated in sustained attention including the medial frontal area and right inferior frontal gyrus (salience/ventral attention network), right superior frontal gyrus (frontoparietal and default mode network), and bilateral superior temporal gyrus (somatomotor network), particularly in performance on tasks, such as the sustained attention to response task (SART) (Manly et al., 2003; Morandini et al., 2020). The reliance of late-developing cognitive functions such as sustained attention on predominantly frontal regions and networks tallies with current understanding of regional brain development, with frontal regions developing last and often well into adolescence and early adulthood (Morgan et al., 2018). Many of the studies that have examined the relationship between sustained attention and neural development do so by using both normative and clinical groups, such as those with ADHD.

Individuals with ADHD commonly experience deficits in sustained attention (McAvinue et al., 2015; Tamm et al., 2012) with performance 1-3 years behind typically developing peers over childhood and adolescence (Thomson et al., 2020). There also are known differences in brain function in those with ADHD compared with controls (e.g., within the default mode network and between default, salience, and frontoparietal networks; Gao et al., 2019; Sutcubasi et al., 2020). Interestingly, many of these networks showing differences between ADHD and typically developing groups also have been linked to sustained attention (O’Halloran et al., 2018; Rosenberg et al., 2016). For example, task-based functional magnetic resonance imaging (fMRI) studies comparing ADHD and control groups have found differences in within-network connectivity of the default mode, salience, and frontoparietal networks explaining deficits in attention maintenance in ADHD (11-18 years, *N* = 40-60: Christakou et al., 2013; Norman et al., 2017). Altered between-network connectivity during a task has also been linked to impaired sustained attention in children with ADHD, such as from motor to frontoparietal and limbic networks (14 years, *N* = 60: O’Halloran et al., 2018). A handful of cross-sectional resting state fMRI studies also have examined how intrinsic functional connectivity relates to sustained attention. Key work by Rosenberg et al. (2016) found two main patterns of connectivity which predicted sustained attention performance in adults (e.g., with connections between cerebellar and frontoparietal networks predicting worse sustained attention, and connections from cerebellar to limbic, motor, and visual networks predicting better sustained attention). These sets of connections also related to ADHD symptoms in 113 children aged 8-16 years. Zepf et al. (2019) described between-network connectivity in a 14-node attention network, which related to ADHD symptoms and general attention problems [9-14 years, *N* = 38], particularly implicating connectivity between salience/ventral attention and visual networks, within the salience network, and between dorsal attention and default mode networks. While connectivity within known functional networks may be pertinent to sustained attention performance (Christakou et al., 2013; Norman et al., 2017), the degree of functional connectivity between networks also may be influential. If this is the case, changes in between-network connectivity may explain improvements in sustained attention over childhood and adolescence, and differences in this process could explain attention deficits in ADHD; however, no studies currently use longitudinal data to examine the links between functional connectivity and sustained attention over the transition to adolescence.

### Aims and Hypotheses

This study employed longitudinal resting state fMRI data to examine changes in within- and between-network functional connectivity over late childhood and early adolescence (Aim 1). It also examined differences in functional connectivity in participants with ADHD relative to typically developing controls (Aim 2). Finally, the study examined how within- and between-network functional connectivity relates to the development of sustained attention, and whether differences in functional connectivity can explain attention deficits in those with ADHD (Aim 3). We hypothesized that: (a) with age, children will show an increase in within-network functional connectivity and a decrease in between-network functional connectivity; (b) children with ADHD will show a delay or altered trajectory of functional connectivity development compared with control children; (c) participants with more segregated neural networks at rest will show better sustained attention performance, and delays or differences in the segregation process will explain the poorer sustained attention performance in children with ADHD compared to control peers of the same age.

## Methods

### Participants

The Neuroimaging of the Children’s Attention Project (NICAP) (Silk et al., 2016) represents a subsample of the Children’s Attention Project (CAP), which is a community-based, longitudinal study, approved by The Royal Children’s Hospital Melbourne Human Research Ethics Committee (Sciberras et al., 2013). In CAP, children aged 6-8 years children were first screened across 43 socioeconomically diverse schools in Melbourne, Australia, using the Conners 3 ADHD Index (Conners, 2008) to identify participants at risk of ADHD and age- and sex-matched non-ADHD controls. Participants with an intellectual disability (IQ < 70), serious medical condition (e.g., kidney disease), genetic disorder, moderate-severe sensory impairment, or neurological disorder were excluded. ADHD or non-ADHD status was confirmed using a diagnostic interview with the parent (Diagnostic Interview Schedule for Children IV [DISC-IV]; Shaffer et al., 2000). Children were followed up at 18-month intervals and invited to join NICAP substudy at the third wave of CAP (9-11 years old).

NICAP assessments were undertaken at the Murdoch Children’s Research Institute, Melbourne, Australia following written informed consent from the parent. Complete details regarding recruitment and study protocol are provided in Sciberras et al. (2013) and Silk et al. (2016). Cognitive assessments and MRI scans were repeated at 18-month intervals, allowing for up to three timepoints of data per participant at ages 9.4-11.9, 10.7-13.4, and 12.1-14.5, respectively. At the first assessment, the DISC-IV parent interview (Shaffer et al., 2000) was repeated to identify any participants with late-onset ADHD symptoms. Participants meeting criteria for ADHD at either baseline or first assessment were included in the ADHD group. The current study comprised 173 individuals (88 ADHD, 85 Control) with complete demographic and MRI data at a minimum of one timepoint.

### Procedure and Measures

#### Demographic Measures

The Wechsler Abbreviated Scale of Intelligence vocabulary and matrix reasoning subtests (Wechsler, 1999) provided an index Intelligence quotient (IQ) at baseline of CAP (6-8 years old). The Socio-economic Indexes for Areas (SEIFA, Index of Relative Socio-Economic Advantage and Disadvantage; Australian Bureau of Statistics, 2013) used the child’s postcode of residence at timepoint one of NICAP to determine socioeconomic status (SES) where higher scores indicated less disadvantage. Medication use information was collected from parents or caregivers at the end of assessment. Twenty-eight participants with ADHD were taking ADHD medication at least one assessment day: 18 at wave 1 (11 methylphenidate, 5 combined methylphenidate and clonidine, 2 atomoxetine), 19 at wave 2 (14 methylphenidate, 3 combined methylphenidate and clonidine, 2 atomoxetine), 10 at wave 3 (8 methylphenidate, 1 combined methylphenidate and clonidine, 1 lisdexamfetamine).

#### MRI Acquisition

Before MRI scanning, participants underwent a mock training scan in which they practiced lying still and listened to sound recordings of a range of MRI scanner noises to familiarize children with common MRI sounds. This has been shown to significantly improve scan completion rates and reduce in-scanner head motion (Simhal et al., 2021). MRI images were acquired using a 3-Tesla Siemens research scanner (Erlangen, Germany) at a single site. Waves 1 and 2 were collected on a TIM Trio scanner; wave 3 was collected after an upgrade to a MAGNETOM Prisma scanner (potential effects of scanner upgrade are addressed in Table S1). T1-weighted structural images were acquired using a multi-echo magnetization prepared rapid gradient echo sequence (176 slices; TR = 2.53 s; TE = 1.77, 3.51, 5.32, 7.20 ms; TI = 1.26 s; flip angle = 7°; FOV = 230 mm; base resolution = 256; slice thickness = 0.9 mm). Resting state scans were acquired over 6 minutes 33 seconds (60 slices; TR = 1.5 s; TE = 33 ms; multi-band factor = 3; flip angle = 85°; FOV = 255 mm; base resolution = 104; slice thickness = 2.5 mm), followed by two 24 second sequences acquired with reverse phase encoding directions (TR = 3.98 s; TE = 33 ms; multiband factor = 1; flip angle = 85°; FOV = 255 mm; base resolution = 104; slice thickness = 2.5 mm; phase encoding direction = reversed), while participants stared at a white fixation cross.

#### MRI Preprocessing

Given the potential for high motion in children with and without ADHD, several steps were taken to attend to head motion. First, MRIQC version 0.14.2 (Esteban et al., 2017) was run for initial data quality control. Framewise displacement values were extracted to identify any resting state scan with extreme head motion, defined as more than 50% of volumes with framewise displacement above 0.5 mm, leading to the initial exclusion of 19 scans across the three timepoints. Preprocessing was then performed using fMRIPrep 1.5.8 (Esteban et al., 2019; Esteban et al., 2020a), which is based on Nipype 1.4.1 (Esteban et al., 2020b; Gorgolewski et al., 2011). Briefly, preprocessing included susceptibility distortion correction, co-registration to T1w image using boundary-based registration with nine degrees of freedom and resampling in FSL’s MNI 152 nonlinear 6th Generation Asymmetric space. Removal of motion artifacts was completed using independent component analysis with 100 components (ICA-AROMA; Pruim et al., 2015) on the preprocessed images in MNI space timeseries after removal of non-steady state volumes and spatial smoothing with an isotropic, Gaussian kernel of 6 mm full-width half-maximum. ICA-AROMA is an automated, high accuracy and robust motion correction approach which decomposes imaging data into components reflecting brain activity or structured noise, so that isolated noise components can be removed. ICA-AROMA has been found to significantly decrease correlations between head motion and functional connectivity (Pruim et al., 2015). Furthermore, a recent study compared several motion correction strategies on a range of benchmarks, recommending ICA-AROMA without global signal regression for studies of network organization due to its ability to decrease head motion and connectivity correlations while maintaining high network identifiability and low distance dependence (Parkes et al., 2018). Further fMRIPrep preprocessing details can be found in 18. Final preprocessing steps were undertaken in the CONN toolbox version 19c (Whitfield-Gabrieli & Nieto-Castanon, 2012), including regression of white matter and cerebrospinal fluid confounds (including first derivatives), linear detrending, and band-pass filtering (0.008-0.09 Hz).

#### Network Parcellation

**Fig. 1:**
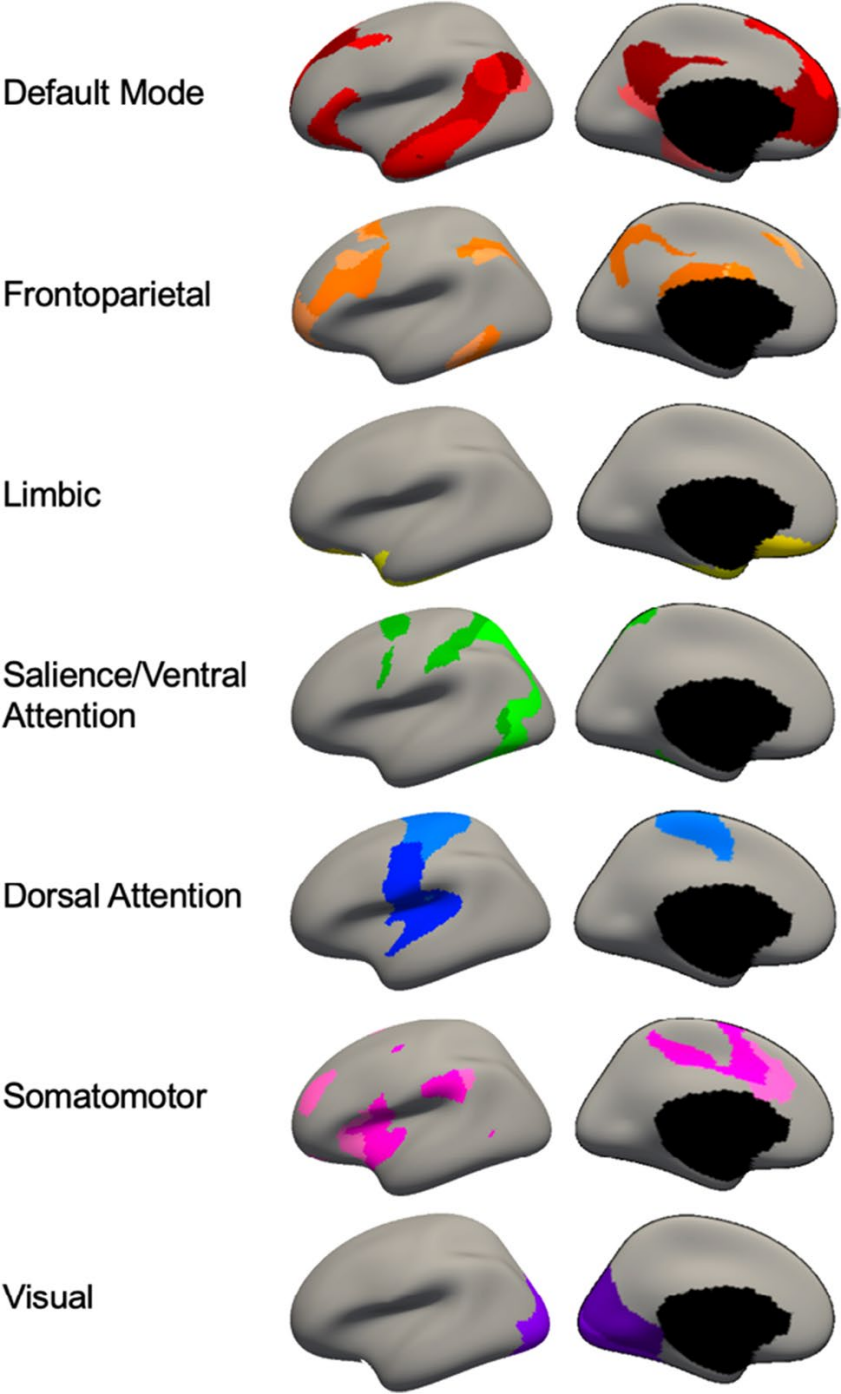
Networks of Yeo et al. (2011) on inflated brain from Freesurfer (7.2.0) showing the mapping from 17 subnetworks to 7 major networks from Baker et al. (2014)

Networks selected for the current study were those from Yeo et al. (2011). Timeseries were averaged over voxels in each ROI (based on 17-network scheme) and correlated between each pair of nodes using Pearson’s correlation analysis. Correlation coefficients were Fisher’s Z-transformed and extracted for further analysis. For interpretability and following previous research (Baker et al., 2014) each of the 17 networks were classified as subregions of the 7 resting state networks [default mode (DMN), dorsal attention (DAN), frontoparietal control (FPN), limbic (LIM), salience ventral attention (SVAN), somatomotor (SOM), and visual (VIS)] allowing for the calculation of within- and between-network functional connectivity. A visual representation of the subnetworks, which comprised each of the 7 networks is displayed in Fig. 1.

#### Sustained Attention

Sustained attention was assessed at each timepoint using the sustained attention to response task (SART) Fixed version (Manly et al., 2003) in E-prime. Participants were shown the digits 1-9 sequentially and asked to press a computer key as quickly as possible following each digit except 3. The task included 225 trials in total (i.e., each digit was displayed 25 times) and lasted 5.5 minutes (see Fig. S1 for further details). Previous SART research has evidenced its strong test-retest reliability over short timescales (Johnson et al., 2008; Robertson et al., 1997), concurrent validity in discriminating between control participants and those with ADHD (Johnson et al., 2007; Salomone et al., 2016), and ecological validity with real-world sustained attention failures (Smilek et al., 2010). SART variable used for the current study was ex-Gaussian parameter *tau* of the response time distribution. Parameters were estimated in MATLAB (Version 9.2) from the raw response times of “go” trials (digits 1-2 or 4-9) at least 100ms long using an iterative maximum likelihood estimation (code available from Lacouture & Cousineau, 2008).

### Statistical Analysis

Changes in within- and between-network functional connectivity and associations with sustained attention were assessed using linear mixed effects (LME) models via the *lme* function of the *nlme* package (version 3.1-148; Pinheiro et al., 2007) in R version 3.6.1. Models were run separately within each network and for each between-network pair. A progressive model fitting approach was used to compare LME models of increasing complexity until the most parsimonious fit was reached. Fit statistics included Akaike information criterion (AIC), Bayesian information criterion (BIC), and log-likelihood ratio test (LRT). Following recent reviews and simulation studies describing the merits of a compound fit statistics approach (Lewis et al. 2011; Vrieze 2012), the final model was selected when comparing the current and next most complex model there was no significant improvement in fit according to the LRT, and both the AIC and BIC were lower in the current model. LME models first aimed to map the development of functional connectivity with age and the potential interaction between age and group by comparing the following successive models:

1. Functional connectivity ~ Group + Sex
2. Functional connectivity ~ Group + Sex + Age
3. Functional connectivity ~ Group + Sex + Age + Age x Group

Existing literature suggests an association between age and diagnostic group in sustained attention development (Thomson et al., 2020). The current study extends such existing models to investigate whether within- and between-network functional connectivity could help to better model attention development. Tested models were as follows:

1. Sustained attention ~ Age + Group + Sex
2. Sustained attention ~ Age + Group + Sex + Functional connectivity
3. Sustained attention ~ Age + Group + Sex + Functional connectivity x Age
4. Sustained attention ~ Age + Group + Sex + Functional connectivity x Age + Functional connectivity x Age x Group

In all models age was grand mean centered, group was classified as either ADHD or non-ADHD control, and a continuous autocorrelation structure for age was included. The continuous autocorrelation structure for age takes into account that values from the same individual over time are positively correlated, but the degree of correlation depends on the length of time between a participant’s assessments. Sex was included as a covariate in all models following research demonstrating sex differences in both functional connectivity (Gur & Gur, 2016) and sustained attention (Gur et al., 2012). Model B4 included all lower order interactions (Functional connectivity x Age, Functional connectivity x Group, Age x Group), although Functional connectivity x Age and Functional connectivity x Age x Group interactions were of primary interest. All models included a random intercept for each subject. After final models were determined, these models were compared to a model additionally including a random age slope for each participant (where applicable) using the model comparison approach, and if fit improved random slopes were additionally included in the final model. Maximum likelihood (ML) estimation was used for initial model comparisons, but restricted maximum likelihood (REML) estimation used to provide most accurate final model parameters (Greven & Kneib, 2010; Wolfinger, 1993). Once final model parameters were estimated, FDR correction was applied to functional connectivity and sustained attention models separately to determine significance of coefficients after accounting for multiple comparisons.

With head motion linked to age and ADHD diagnosis, inclusion as a covariate likely results in an underestimation of effects of interest (Dosenbach et al., 2017; Kong et al., 2014; Thomson et al., 2021). However, given the potential for motion in our sample and to ensure confidence in findings, models were rerun in two motion-matched subsamples following the matching approach of Satterthwaite et al. (2013). Further details of these subsamples can be found in 18. Because a small number of participants with ADHD were taking medication, two relevant supplementary analyses were provided: 1) rerunning models with ADHD medication use as a covariate; 2) rerunning models in motion-matched subsamples which included only participants without ADHD medication use.

## Results

### Participant Characteristics

**Fig. 2:**
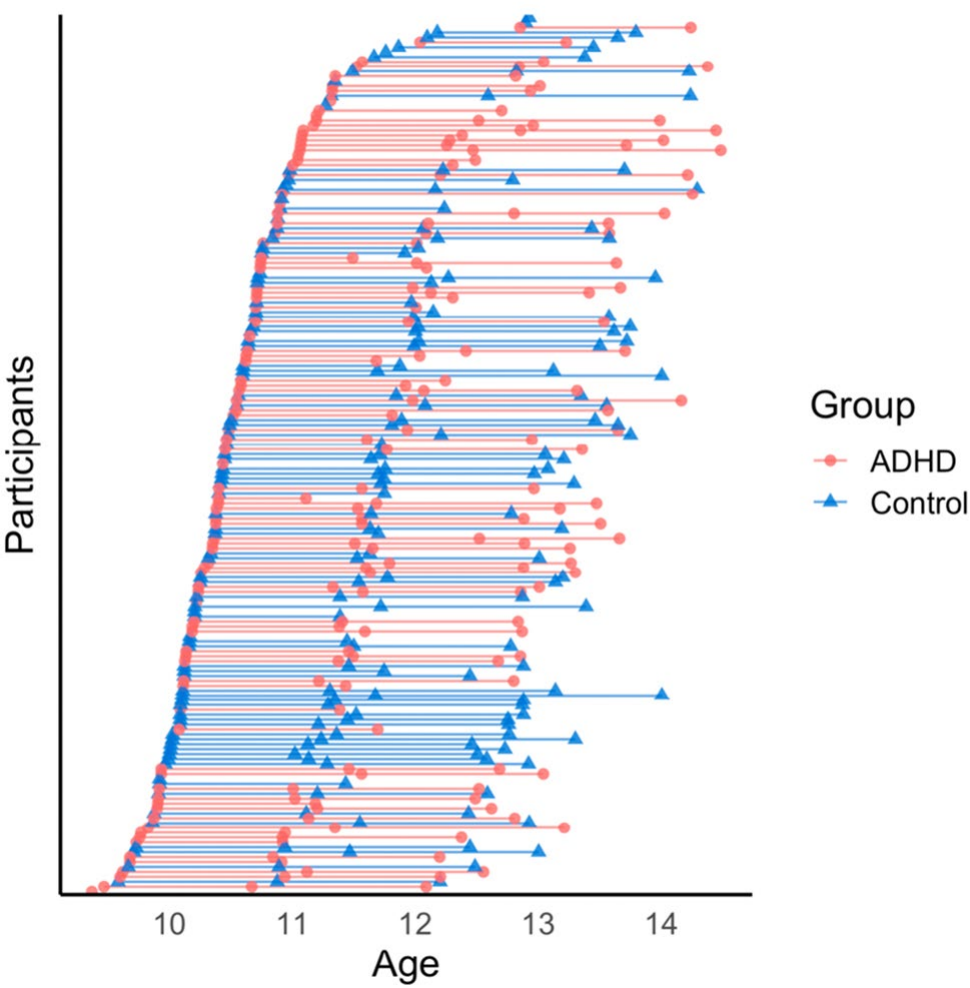
Longitudinal data available in the ADHD and control groups

The final sample comprised 173 participants with complete demographic and functional MRI data for least one wave, with 83% having two or more scans (see Fig. 2 for visualization and Table 1 for sample characteristics). Within this sample, 159 participants also had valid sustained attention data that was used for sustained attention models (sample characteristics in Table S2).

**Table 1:** Sample characteristics

	ADHD	Control	Test of significance
Total data points [% male]	197 (76%)	201 (55%)	-
Participants wave 1 [% male]	65 (77%)	77 (56%)	-
Participants wave 2 [% male]	77 (75%)	70 (53%)	-
Participants wave 3 [% male]	55 (75%)	54 (57%)	-
Age wave 1 [mean (SD)]	10.4 (0.5)	10.4 (0.4)	*t*(140) = −0.03, *p* = 0.978, *d* = −0.01
Age wave 2 [mean (SD)]	11.7 (0.6)	11.8 (0.5)	*t*(145) = −0.88, *p* = 0.379, *d* = −0.15
Age wave 3 [mean (SD)]	13.2 (0.6)	13.2 (0.5)	*t*(107) = 0.33, *p* = 0.744, *d* = 0.06
% Male participants	88 (77%)	85 (56%)	***χ***^**2**^**(1,173) = 8.47,** ***p*** **= 0.004,** ***V =*** **0.22**
IQ [mean (SD)]	96 (14)	103 (13)	***t*(170) = -3.81,** ***p*** **< 0.001,** ***d =*** **−0.58**
SES [mean (SD)]	1021 (39)	1019 (47)	*t*(171) = 0.32, *p* = 0.748, *d* = 0.05
DISC-IV inattention symptoms [median (IQR)]	7.0 (1.0)	0.0 (2.0)	***U =*** **6979,** ***p*** **< 0.001,** ***r*** **= 0.87**
DISC-IV hyperactivity-impulsivity symptoms [median (IQR)]	5.0 (2.0)	0.0 (1.0)	***U =*** **6838,** ***p <*** **0.001,** ***r*** **= 0.83**
Internalizing disorder [count (% group)]	21 (24%)	8 (10%)	***χ***^**2**^**(1,172) = 6.30,** ***p*** **= 0.012,** ***V =*** **0.19**
Externalizing disorder [count (% group)]	45 (51%)	8 (10%)	***χ***^**2**^**(1,172) = 34.07,** ***p*** **< 0.001,** ***V =*** **0.45**
Framewise displacement [median (IQR)]	0.13 (0.13)	0.10 (0.08)	***U =*** **22752,** ***p*** **= 0.002,** ***r*** **= 0.19**

*Note.* Tests of significance employed were independent samples t-test, chi-squared test, and Mann-Whitney *U* test where appropriate. Intelligence quotient (IQ) based on Wechsler Abbreviated Scale of Intelligence (Wechsler, 1999). Socioeconomic status (SES) based on SEIFA (Index of Relative Socio-Economic Advantage and Disadvantage; Australian Bureau of Statistics, 2013). ADHD symptom levels and presence of internalizing (depression, dysthymia, separation anxiety disorder, social phobia, generalized anxiety disorder, posttraumatic stress disorder, obsessive-compulsive disorder, hypomania or manic episode) or externalizing problems (oppositional defiant disorder or conduct disorder) based on the Diagnostic Interview Schedule for Children IV (DISC-IV) at baseline (Shaffer et al., 2000). Framewise displacement (in-scanner head motion; Jenkinson et al., 2002) correlated with age across the sample, *r*_*s*_ = −0.18, *p* < 0.001. Bold indicates significant group difference. SD = standard deviation

### Functional Connectivity Development

In the preliminary analysis of potential age-related changes in functional connectivity (Aim 1), the inclusion of age as an independent variable improved models of functional connectivity for the majority (19/28) of possible network connections (Table 2). See Table S3 for fit statistics leading to selected models. Following FDR correction, functional connectivity was found to have significantly decreased with age between the frontoparietal network and each of the salience/ventral attention, visual and limbic networks, as well as between the default mode and limbic, and salience/ventral attention and visual networks, after accounting for clinical group and sex. Within these models, males had greater functional connectivity than females for 4 network connections (salience/ventral attention-visual, default mode-limbic, default mode-visual, and dorsal attention-visual). No other sex differences were evident. For within network connectivity, models indicated a significant age effect on functional connectivity within the frontoparietal and default mode networks (decreasing across age), although note this did not survive FDR correction. No other age effects on within-network connectivity were observed.

**Table 2:** Linear mixed effects models of network functional connectivity development

Connection of interest			Variable [regression coefficient (standard error), FDR-corrected *p* value]				
Network 1	Network 2	Model form	(Intercept)	Age	Sex	Group	Age x Group
Default mode	Default mode	A3	0.76 (0.04)***	-0.04 (0.02)^	0.04 (0.04)	-0.03 (0.04)	0.03 (0.03)
Default mode	Dorsal attention	A3	0.51 (0.04)***	-0.03 (0.02)	0.07 (0.04)	-0.01 (0.04)	0.04 (0.02)
Default mode	Frontoparietal	A3	0.54 (0.04)***	-0.04 (0.02)^	0.07 (0.04)	-0.01 (0.04)	0.04 (0.02)
Default mode	Limbic	A2	0.36 (0.04)***	-0.03 (0.01)*	0.11 (0.04)*	-0.03 (0.04)	-
Default mode	Salience/ventral attention	A3	0.52 (0.04)***	-0.04 (0.02)	0.03 (0.04)	-0.02 (0.04)	0.03 (0.03)
Default mode	Somatomotor	A3	0.53 (0.03)***	-0.02 (0.02)	0.03 (0.03)	-0.01 (0.03)	0.02 (0.02)
Default mode	Visual	A1	0.68 (0.03)***	-	0.09 (0.03)*	0.02 (0.03)	-
Dorsal attention	Dorsal attention	A1	0.51 (0.05)***	-	0.06 (0.04)	0.02 (0.04)	-
Dorsal attention	Frontoparietal	A2	0.62 (0.03)***	-0.02 (0.01)	0.02 (0.03)	-0.02 (0.03)	-
Dorsal attention	Limbic	A3	0.71 (0.05)***	-0.04 (0.02)^	0.09 (0.05)	-0.02 (0.04)	0.05 (0.03)
Dorsal attention	Salience/ventral attention	A3	0.83 (0.04)***	-0.04 (0.02)	0.05 (0.04)	0.00 (0.04)	0.03 (0.03)
Dorsal attention	Somatomotor	A2	0.52 (0.03)***	-0.01 (0.01)	0.07 (0.03)^	0.00 (0.03)	-
Dorsal attention	Visual	A1	0.57 (0.04)***	-	0.09 (0.04)*	0.03 (0.03)	-
Frontoparietal	Frontoparietal	A2	0.42 (0.04)***	-0.03 (0.01)	0.07 (0.04)	-0.03 (0.04)	-
Frontoparietal	Limbic	A3	0.48 (0.04)***	-0.05 (0.02)**	0.06 (0.04)	-0.04 (0.04)	0.03 (0.02)
Frontoparietal	Salience/ventral attention	A3	0.61 (0.04)***	-0.04 (0.02)*	0.05 (0.04)	-0.01 (0.03)	0.04 (0.02)
Frontoparietal	Somatomotor	A1	0.49 (0.03)***	-	0.05 (0.03)	-0.03 (0.03)	-
Frontoparietal	Visual	A3	0.55 (0.04)***	-0.04 (0.02)*	0.08 (0.04)^	0.02 (0.04)	0.05 (0.02)^
Limbic	Limbic	A1	0.56 (0.04)***	-	0.01 (0.04)	0.01 (0.03)	-
Limbic	Salience/ventral attention	A3	0.54 (0.04)***	-0.03 (0.02)	0.04 (0.04)	-0.02 (0.04)	0.03 (0.02)
Limbic	Somatomotor	A2	0.51 (0.04)***	-0.02 (0.01)^	0.05 (0.04)	-0.01 (0.04)	-
Limbic	Visual	A3	0.56 (0.03)***	-0.03 (0.01)^	0.07 (0.03)^	0.01 (0.03)	0.04 (0.02)^
Salience/ventral attention	Salience/ventral attention	A1	0.78 (0.05)***	-	0.06 (0.05)	-0.03 (0.05)	-
Salience/ventral attention	Somatomotor	A3	0.41 (0.04)***	-0.03 (0.02)	0.08 (0.04)^	-0.01 (0.04)	0.04 (0.03)
Salience/ventral attention	Visual	A3	0.8 (0.03)***	-0.04 (0.01)*	0.08 (0.03)*	0.01 (0.03)	0.05 (0.02)*
Somatomotor	Somatomotor	A1	0.49 (0.04)***	-	0.06 (0.04)	0.02 (0.04)	-
Somatomotor	Visual	A1	0.52 (0.04)***	-	0.07 (0.03)	0.01 (0.03)	-
Visual	Visual	A1	0.98 (0.03)***	-	0.02 (0.03)	-0.01 (0.03)	-

*Note.* Bold identifies significant independent variable following false discovery rate (FDR) correction from the Benjamini-Hochberg method (Benjamini & Hochberg, 1995). See equations for final models A1-3 in *Methods*. All models include a random intercept; models with age variable include a random age slope. *FDR-corrected *p* < 0.050; **FDR-corrected *p* < 0.010; ***FDR-corrected *p* < 0.001; ^*p* < 0.050 before FDR-correction only. Exact *p* values provided in Table S5. Hyphen indicates variable was not involved in best fitting model

**Fig. 3:**
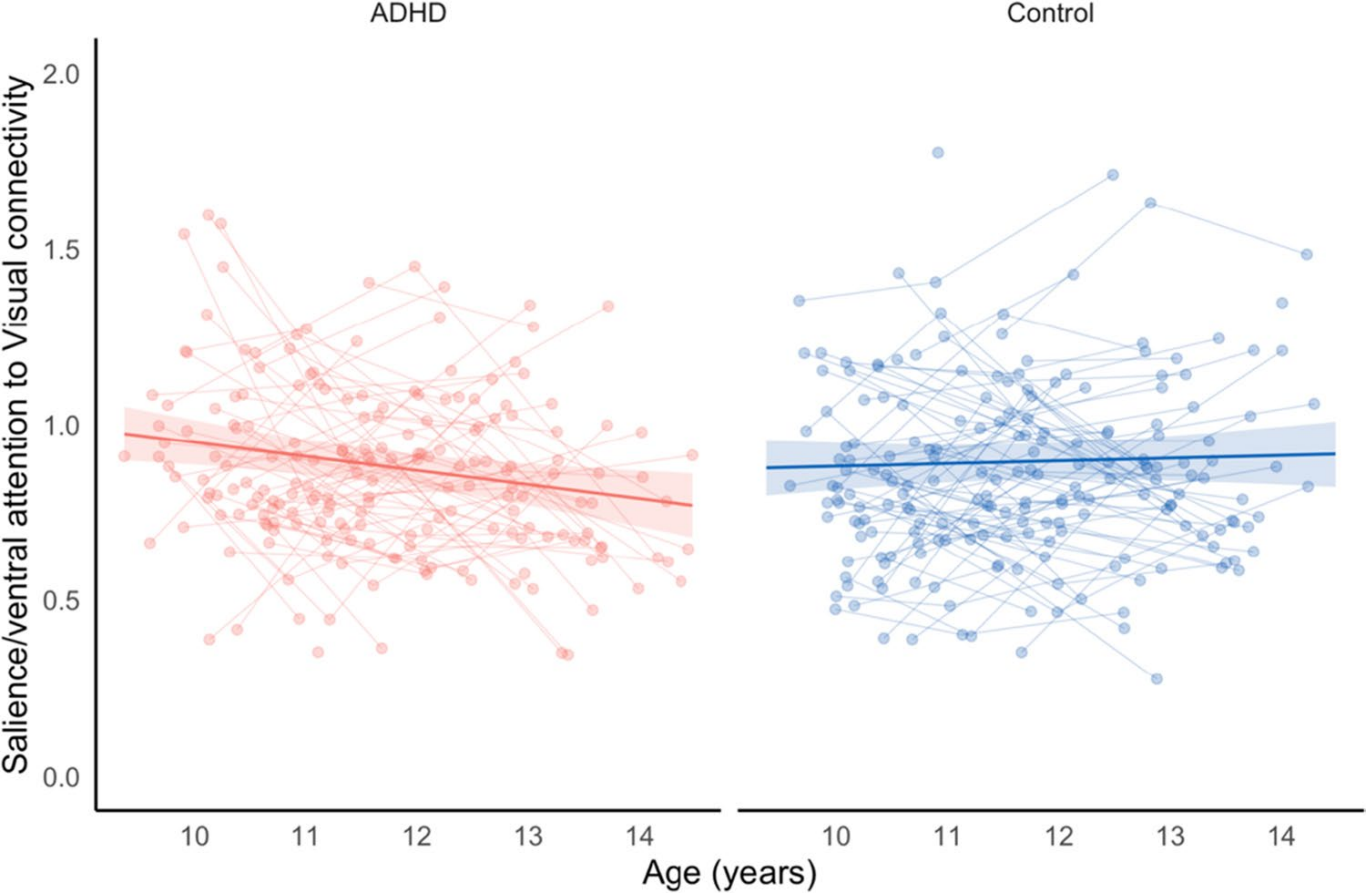
Change in functional connectivity between the salience/ventral attention network and visual network in the ADHD and Control groups over 9-14 years

To examine group differences in connectivity development (Aim 2), the inclusion of an interaction between age and group significantly improved model fit for 10 network connections. Following FDR correction, the change in functional connectivity across age between the salience/ventral attention and visual network, differed between the ADHD and control groups. While controls demonstrated minimal change in functional connectivity between the salience/ventral attention and visual networks over the age span, connectivity in children with ADHD decreased, dropping to levels below typical controls (Fig. 3). No other interactions were significant. There were no significant main effects of group. See Tables S9 and S11 for supplementary subgroup analyses.

### Sustained Attention Development

For the sustained attention models (Aim 3), we found that functional connectivity in 11 of 28 possible network connections improved models of sustained attention development via the ex-Gaussian measure tau (see Table 3, and Table S4 for fit statistics leading to selected models). Notably, these network connections included connectivity from the frontoparietal network to all other networks. It additionally included connections from the default mode to salience, default mode to limbic, dorsal attention to limbic, and salience to somatomotor networks.

**Table 3:** Linear mixed effects models of sustained attention development

Connection of interest			Variable [regression coefficient (standard error), FDR-corrected *p* value]						
Network 1	Network 2	Model form	(Intercept)	Age	Sex	Group	FC	FC x Age	FC x Age x Group
Default mode	Frontoparietal	B3	116.62 (9.14)***	-12.61 (3.59)**	-0.58 (10.68)	23.51 (10.14)*	3.58 (1.01)**	2.54 (0.84)**	-
Default mode	Limbic	B3	117.72 (9.12)***	-12.38 (3.57)**	-0.81 (10.66)	23.46 (10.12)*	2.76 (1.23)*	3.10 (1.06)**	-
Default mode	Salience/ventral attention	B3	117.43 (9.14)***	-13.37 (3.60)**	-0.47 (10.66)	23.51 (10.12)*	2.14 (1.46)	4.09 (1.18)**	-
Dorsal attention	Frontoparietal	B4	118.50 (9.27)***	-14.99 (5.30)*	-0.04 (10.66)	18.48 (10.47)	7.67 (2.51)**	10.53 (2.12)***	-7.10 (3.13)*
Dorsal attention	Limbic	B3	115.07 (9.20)***	-14.22 (3.77)**	-0.87 (10.60)	23.7 (10.07)*	4.74 (2.20)*	3.95 (1.83)*	-
Frontoparietal	Frontoparietal	B3	116.17 (9.05)***	-13.37 (3.56)**	-1.28 (10.53)	23.89 (10.00)*	6.07 (2.35)*	5.20 (2.01)*	-
Frontoparietal	Limbic	B3	117.03 (9.11)***	-13.20 (3.58)**	-0.77 (10.63)	23.58 (10.10)*	3.45 (1.48)*	4.29 (1.26)**	-
Frontoparietal	Salience	B3	116.80 (9.13)***	-13.47 (3.61)**	-0.52 (10.63)	23.61 (10.09)*	2.75 (1.48)^	3.60 (1.20)**	-
Frontoparietal	Somatomotor	B3	116.75 (9.13)***	-13.59 (3.62)**	-0.57 (10.63)	23.38 (10.09)*	3.81 (1.98)^	4.68 (1.66)*	-
Frontoparietal	Visual	B3	116.32 (9.17)***	-14.15 (3.66)***	-0.65 (10.65)	23.79 (10.12)*	3.48 (1.85)^	4.77 (1.53)**	-
Salience/ventral attention	Somatomotor	B3	155.03 (9.07)***	-15.22 (3.64)***	-1.08 (10.53)	23.72 (10.00)*	8.43 (2.58)**	8.65 (2.11)***	-

*Note*. Bold identifies significant independent variable following false discovery rate (FDR) correction from the Benjamini-Hochberg method (Benjamini & Hochberg, 1995). See equations for final models B1-4 in *Methods*. All models include a random intercept and random age slope. Model including dorsal attention to frontoparietal functional connectivity (FC) also contained lower order interactions: FC x Group, −6.40 (3.73), *p* = 0.111; and Age x Group, −0.89 (7.35), *p* = 1.000. *FDR-corrected *p* < 0.050; **FDR-corrected *p* < 0.010; ***FDR-corrected *p* < 0.001; ^*p* < 0.050 before FDR-correction only. Exact *p* values provided in Table S6. Hyphen indicates variable was not involved in best fitting model

**Fig. 4:**
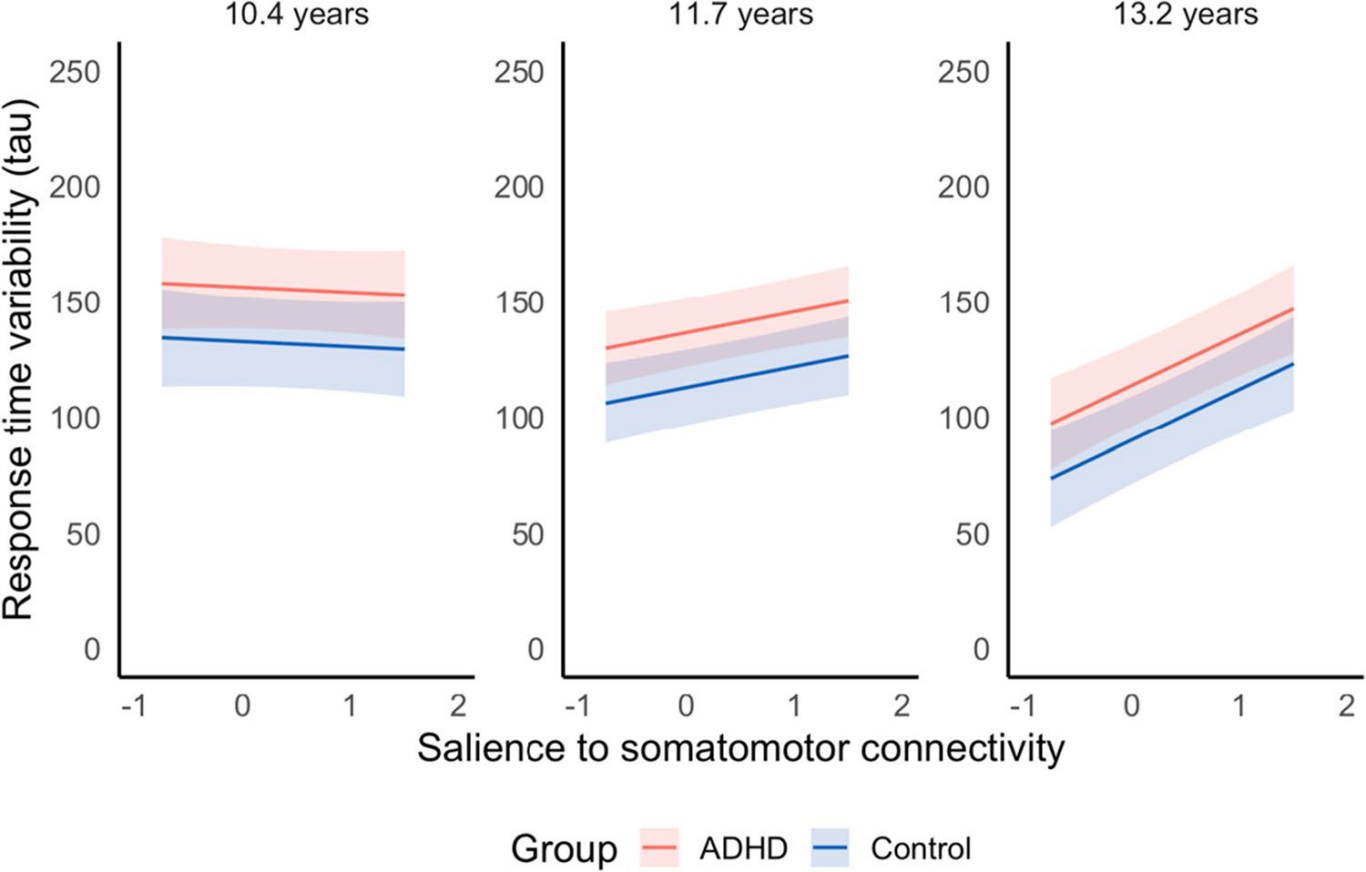
Effect of functional connectivity between the salience/ventral attention network and somatomotor network on change in sustained attention. Greater tau represents higher response time variability (worse sustained attention). For visualization purposes, relationship between sustained attention and functional connectivity depicted at the mean age for each wave

In all 11 models, there was a main effect of age and group on sustained attention; children generally improved in sustained attention with age, and those with ADHD had consistently worse attention across the age range. There also was a significant interaction between age and connectivity. Whereas in late childhood, the degree of connectivity between these 11 network pairs had minimal influence on attention performance, by early adolescence those with lower connectivity between these networks showed significantly better sustained attention ability on average compared with those with high connectivity and compared to performance in previous years. See Fig. 4 for a visualization of this interaction effect, taking the salience/ventral attention and somatomotor network pair as an example. This relationship between network connectivity and sustained attention was comparable in both the ADHD and control groups, although participants with ADHD showed consistently worse sustained attention via the group main effect.

**Fig. 5:**
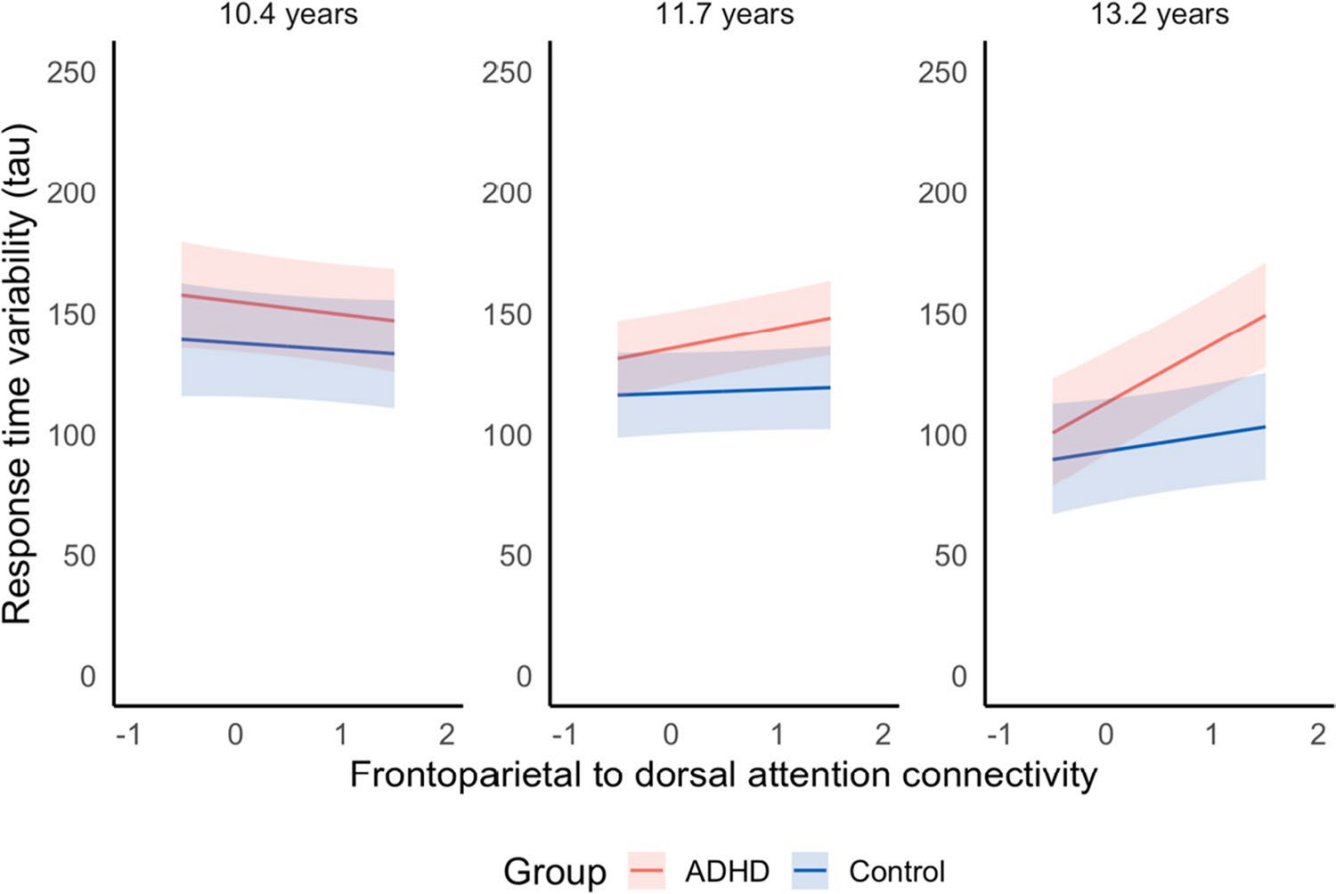
Effect of functional connectivity between the frontoparietal network and dorsal attention network on change in sustained attention. Greater tau represents higher response time variability (worse sustained attention). For visualization purposes, relationship between sustained attention and functional connectivity depicted at the mean age for each wave

Finally, regarding Aim 3, following FDR correction there was a significant three-way interaction between age, group and connectivity between the dorsal attention and frontoparietal networks (Fig. 5). Connectivity between the frontoparietal and dorsal attention networks did not relate to sustained attention performance in children with ADHD. By adolescence, those with ADHD with low connectivity between the dorsal attention and frontoparietal networks showed sustained attention performance that was both comparable to controls and significantly better compared with same-aged adolescents with ADHD with high connectivity between these networks. Participants in the ADHD group with low connectivity between these networks also were estimated to have improved sustained attention on average compared to previous years. Meanwhile, adolescents with ADHD with high connectivity between the dorsal attention and frontoparietal networks showed worse sustained attention (compared to controls and adolescents with ADHD with low connectivity between these networks) and minimal improvements in attention on average since childhood. Controls improved in sustained attention over the late childhood and early adolescent period. However, unlike in the ADHD group, the degree of functional connectivity between the frontoparietal and dorsal attention network did not influence sustained attention performance at any of the studied ages in controls. No other models included a three-way interaction. Effects were consistent after rerunning models in supplementary analyses (Tables S10 and S12).

## Discussion

This study assessed changes in functional connectivity over the mid-to-late childhood to early adolescent period and examined how levels of connectivity influence sustained attention development in both typical controls and ADHD. Whereas in late childhood the degree of connectivity between networks, such as the frontoparietal and salience to somatomotor, appeared to have little influence on attention ability, by early adolescence those with low connectivity had developed better attention performance, suggesting a change over time in the importance of between-network connectivity for sustained attention. Lowered functional connectivity between the frontoparietal and dorsal attention network appeared particularly important for attention performance in individuals with ADHD, and may be a valuable focus of future attention intervention research.

### Connectivity Underlying Attention Development

Resting state network connectivity between 11 network pairs was associated with sustained attention development. These network pairs included the frontoparietal network to all other networks (although note as main effects only connections with the default mode, dorsal attention, and limbic networks survived FDR correction), as well as default mode to salience and limbic networks, dorsal attention to limbic network, and salience to somatomotor network. In all cases, adolescents with lower connectivity between networks demonstrated better sustained attention ability on average compared with those with higher connectivity and compared with performance in previous years. This shows the importance of the frontoparietal network in sustained attention and matches existing sustained attention literature (Norman et al., 2017). Findings also align with previous work highlighting the importance of network segregation for brain and cognitive development (Fair et al., 2009) but demonstrates for the first time that, rather than being specific to certain network connections, such as frontoparietal to default mode connectivity, sustained attention performance may benefit from the segregation of the frontoparietal network from all other brain networks.

Alongside the frontoparietal network, a strong benefit to sustained attention maturation was seen for adolescents with low connectivity from the salience/ventral attention to both default mode and somatomotor networks. Task-based fMRI has previously found that increasing activation of salience/ventral attention and decreasing activity in the default mode network (i.e., decreased coactivation of default and salience/ventral attention networks) over time supports maintenance of attention (Christakou et al., 2013; Norman et al., 2017). Similarly, greater autonomy of the somatomotor network is thought to assist with cognitive performance (Gu et al., 2015; Power et al., 2011), supported by task-based fMRI literature linking reduced connectivity between motor and prefrontal regions to better sustained attention (O’Halloran et al., 2018).

For both the frontoparietal and salience/ventral attention network findings, although by adolescence the degree of connectivity between networks is associated with better sustained attention, the level of connectivity between these networks did not appear relevant to sustained attention in late childhood. It is known that while greater connectivity is associated with better cognitive performance in early childhood (Bruchhage et al., 2020), the converse appears to be true in adults (Wig, 2017). The transition between childhood and adolescence captured in the current study may represent a period of shift for the general relationship between connectivity and cognitive performance relevant to future longitudinal work with data from early childhood to adolescence. Increasing independence of the frontoparietal network and decreased network coupling of the salience to default mode and somatomotor networks may become increasingly important to sustained attention and facilitate adolescent attention development.

Poorer sustained attention in ADHD is well documented (McAvinue et al., 2015; Tamm et al., 2012). For the connections described above, although lower connectivity in adolescence was related to greater attention development from childhood for both groups, the ADHD group showed worse sustained attention at all timepoints. For frontoparietal to dorsal attention network connectivity, however, there was a difference between the ADHD and control groups in the relationship between connectivity and sustained attention. Reduced functional connectivity between the frontoparietal and dorsal attention networks was associated with greater development of attention in adolescents with ADHD, while the degree of frontoparietal to dorsal attention connectivity did not appear relevant to attention development in controls. Adolescents with ADHD with lower frontoparietal to dorsal attention connectivity also showed comparable attention performance to neurotypical peers. There is currently a limited understanding of what reduced frontoparietal to dorsal attention network connectivity may facilitate in terms of behavior and cognition (Zhou et al., 2018); however, this appears to be an area of difference for ADHD in the connectivity underlying sustained attention. This result presents a possible marker for distinguishing fundamental network connectivity differences that benefit sustained attention in those with ADHD and neurotypical adolescents. Connectivity patterns may specifically differentiate individuals with ADHD with and without sustained attention impairment and could prove advantageous in the development of ADHD-specific training programs.

### Connectivity Changes with Age

Connectivity between the salience/ventral attention and visual network changed minimally in controls over the study period, whereas the ADHD group had decreasing connectivity over time which ultimately fell to below neurotypical levels by early adolescence (although note this result did not survive FDR correction in one of the motion-matched subsamples and must be treated with caution until replicated). Previous functional connectivity work across a range of ages has found evidence of lower long-range functional connectivity and greater general segregation between networks in those with ADHD (Cao et al., 2014; Lin et al., 2014). The current work provides initial evidence of this potential loss of long-range connections during development and may correspond with the loss of structural connections identified in previous diffusion MRI work (Beare et al., 2017). The salience to visual network was not a network connection linked to sustained attention in the current study; however, it is not yet clear why, in principle, children with ADHD often may show greater segregation between networks but worse cognitive performance on a range of tasks (Claesdotter et al., 2018; Mills et al., 2018). It may be that while static functional connectivity provides an initial understanding of the optimal brain states for good cognitive performance, future work needs to look as this in conjunction with dynamic functional connectivity (e.g., changes in the pattern of connectivity within a scan) to get a complete picture of the optimal temporal fluctuations between brain states for attention maintenance.

### Limitations

The current work contributes significant knowledge towards understanding how functional connectivity and attention develop in ADHD-affected and typically developing children however must be considered in the context of the following limitations. First, the current study considered only cortical networks; however, atlases, such as those of Buckner et al. (2011) and more recently Ji et al. (2019), provide a means to confirm initial longitudinal studies of subcortical to cortical connectivity (Van Duijvenvoorde et al., 2019) and link the current attention findings to functional development in subcortical and cerebellar regions (Christakou et al., 2013). Second, this study used linear mixed effects models to follow on from previous sustained attention work, however with limited longitudinal functional connectivity data available, further exploration of potential nonlinear effects is important. Finally, we took several steps to prevent head motion, including conducting mock scanner training prior to each MRI scan (Simhal et al., 2021), excluding participants with extreme head motion, and removing motion artifacts using ICA-AROMA (Pruim et al., 2015) based on recommendations from Parkes et al. (2018). However, there remained head motion measure correlations with functional connectivity in the sample (Table S13) likely due to studying a high motion pediatric and ADHD sample. With the variable age influencing head motion, which in turn influences the outcome variable functional connectivity, the inclusion of head motion as a covariate can create problematic downstream effects and distort model estimates (Wilkinson, 2018). To address this, the current study followed the approach of Satterthwaite et al. (2013) and defined motion-matched and nearly motionless subsamples to confirm results. However, future research adopting further preventative motion strategies, such as increased head protection during scanning, is required to confirm findings.

## Conclusions

Expanding on cross-sectional studies, the current longitudinal work found initial evidence of stronger downward trajectories of functional connectivity within participants with ADHD than controls between networks, such as the salience/ventral attention and visual networks, over the late childhood to early adolescent period. Adolescents with greater segregation of the frontoparietal network from all other networks, and salience/ventral attention to default mode and somatomotor networks, showed greater sustained attention development. For the frontoparietal to dorsal attention networks, adolescents with ADHD with reduced connectivity obtained a level of sustained attention ability matching neurotypical peers. This study contributes significant knowledge towards understanding the relationship between brain and cognitive development and mechanisms by which attention deficits may be ameliorated in individuals with ADHD.

## Supplementary Information

ESM 1 (DOCX 258 kb)

## Data Availability

Data from the Children’s Attention Project cohort are available via Lifecourse: https://lifecourse.melbournechildrens.com/cohorts/cap-and-nicap/. The specific data that support the findings of this study are available on request from the corresponding author.

## Code availability

Data were prepared according to documentation from fMRIPrep (https://fmriprep.org/) and CONN toolbox (https://web.conn-toolbox.org/resources/documentation). All code implemented for analysis in the current study were based on detailed R documentation for linear mixed effects modelling from https://www.rdocumentation.org/packages/nlme/versions/3.1-152/topics/lme and code from Lacouture and Cousineau (2008). Custom code that yielded the findings of the current article are available from the corresponding author upon request.
