# Supplementary material for: Longitudinal maturation of resting state networks: Relevance to sustained attention and attention deficit/hyperactivity disorder
Source: Cogn Affect Behav Neurosci. 2022 Jun 8;22(6):1432–46. doi: 10.3758/s13415-022-01017-9 (PMC9622522; doi:10.3758/s13415-022-01017-9)
Supplement: Supplementary file 1 — (DOCX 258 kb) [file 13415_2022_1017_MOESM1_ESM.docx]

**Supplementary Materials**

**MRI preprocessing**

Provided details of imaging preprocessing are derived from the fMRIPrep boilerplate text.

***Anatomical data preprocessing****.* The T1-weighted (T1w) image was corrected for intensity non-uniformity (INU) with N4BiasFieldCorrection (Tustison et al., 2010), distributed with ANTs 2.2.0 (Avants, Epstein, Grossman, & Gee, 2008), and used as T1w-reference throughout the workflow. The T1w-reference was then skull-stripped with a Nipype implementation of the antsBrainExtraction.sh workflow (from ANTs), using OASIS30ANTs as target template. Brain tissue segmentation of cerebrospinal fluid (CSF), white-matter (WM) and gray-matter (GM) was performed on the brain-extracted T1w using fast (FSL 5.0.9; Zhang, Brady, & Smith, 2001). Volume-based spatial normalization to standard space was performed through nonlinear registration with antsRegistration (ANTs 2.2.0), using brain-extracted versions of both T1w reference and the T1w template. The following template was selected for spatial normalization: FSL's MNI ICBM 152 non-linear 6th Generation Asymmetric Average Brain Stereotaxic Registration Model (MNI152NLin6Asym; Evans, Janke, Collins, & Baillet, 2012).

***Functional data preprocessing.*** For each BOLD run per subject the following preprocessing was performed. First, a reference volume and its skull-stripped version were generated using a custom methodology of fMRIPrep. A B0-nonuniformity map (or fieldmap) was estimated based on two (or more) echo-planar imaging (EPI) references with opposing phase-encoding directions, with 3dQwarp (AFNI 20160207; Cox & Hyde, 1997). Based on the estimated susceptibility distortion, a corrected EPI (echo-planar imaging) reference was calculated for a more accurate co-registration with the anatomical reference. The BOLD reference was then co-registered to the T1w reference using flirt (FSL 5.0.9; Jenkinson, Bannister, Brady, & Smith, 2002; Jenkinson & Smith, 2001) with the boundary-based registration (Greve & Fischl, 2009) cost-function. Co-registration was configured with nine degrees of freedom to account for distortions remaining in the BOLD reference. Head-motion parameters with respect to the BOLD reference (transformation matrices, and six corresponding rotation and translation parameters) are estimated before any spatiotemporal filtering using mcflirt (FSL 5.0.9; Jenkinson et al., 2002).

The BOLD time-series were resampled generating spatially-normalized, preprocessed BOLD runs in MNI152NLin6Asym space. First, a reference volume and its skull-stripped version were generated using a custom methodology of fMRIPrep. Automatic removal of motion artifacts using independent component analysis (ICA-AROMA; Pruim et al., 2015) was performed on the preprocessed BOLD in MNI space time-series after removal of non-steady state volumes and spatial smoothing with an isotropic, Gaussian kernel of 6mm FWHM (full-width half-maximum). Corresponding “non-aggressively” denoised runs were produced after such smoothing used for final analysis. Confounding time-series for framewise displacement (FD) were calculated based on the preprocessed BOLD for each functional run, using implementations in Nipype (following the definitions by Jenkinson et at. 2002). The three global signals are extracted within the CSF, the WM, and the whole-brain masks. Many internal operations of fMRIPrep use Nilearn 0.6.1 (Abraham et al., 2014). For more details of the pipeline, see the section corresponding to workflows in fMRIPrep's documentation (<https://fmriprep.readthedocs.io/en/latest/workflows.html>).

**Scanner Upgrade**

Independent samples t-tests comparing network functional connectivity pre- and post-scanner upgrade revealed no mean differences in functional connectivity for any network pair (p-value range = .067-.664, see Table S1), according with previously published work in this sample finding minimal scanner effects (Vijayakumar et al., 2021). Participants included in the comparison comprised 19 participants from wave 2 (pre-upgrade) and 19 participants from wave 3 (post-upgrade) matched on age, group, sex and head motion. After matching, the mean difference in age was 0.08 years (28 days).

Table S1. Independent samples t-tests comparing network functional connectivity pre- and post-scanner upgrade.

| Connection of interest | | Independent samples t-test | | | |
| --- | --- | --- | --- | --- | --- |
| Network 1 | Network 2 | *t* | df | *p* | Cohen's d |
| Default Mode | Default Mode | 1.46 | 36 | .154 | 0.47 |
| Default Mode | Dorsal Attention | 0.79 | 36 | .437 | 0.26 |
| Default Mode | Frontoparietal | 1.27 | 36 | .212 | 0.41 |
| Default Mode | Limbic | 1.49 | 36 | .145 | 0.48 |
| Default Mode | Salience/Ventral Attention | 1.43 | 36 | .160 | 0.47 |
| Default Mode | Somatomotor | 0.76 | 36 | .452 | 0.25 |
| Default Mode | Visual | 0.82 | 36 | .417 | 0.27 |
| Dorsal Attention | Dorsal Attention | 0.46 | 36 | .648 | 0.15 |
| Dorsal Attention | Frontoparietal | 1.89 | 36 | .067 | 0.61 |
| Dorsal Attention | Limbic | 1.16 | 36 | .256 | 0.38 |
| Dorsal Attention | Salience/Ventral Attention | 0.79 | 36 | .432 | 0.26 |
| Dorsal Attention | Somatomotor | 1.46 | 36 | .153 | 0.47 |
| Dorsal Attention | Visual | 0.95 | 36 | .346 | 0.31 |
| Frontoparietal | Frontoparietal | 1.05 | 36 | .303 | 0.34 |
| Frontoparietal | Limbic | 1.49 | 36 | .144 | 0.48 |
| Frontoparietal | Salience/Ventral Attention | 1.25 | 36 | .220 | 0.41 |
| Frontoparietal | Somatomotor | 1.27 | 36 | .211 | 0.41 |
| Frontoparietal | Visual | 1.14 | 36 | .260 | 0.37 |
| Limbic | Limbic | 1.15 | 36 | .259 | 0.37 |
| Limbic | Salience/Ventral Attention | 0.88 | 36 | .384 | 0.29 |
| Limbic | Somatomotor | 1.32 | 36 | .196 | 0.43 |
| Limbic | Visual | 0.5 | 36 | .617 | 0.16 |
| Salience/Ventral Attention | Salience/Ventral Attention | 0.44 | 36 | .664 | 0.14 |
| Salience/Ventral Attention | Visual | 1.6 | 36 | .119 | 0.52 |
| Salience/Ventral Attention | Somatomotor | 1.05 | 36 | .301 | 0.34 |
| Somatomotor | Somatomotor | 1.05 | 36 | .302 | 0.34 |
| Somatomotor | Visual | 0.48 | 36 | .634 | 0.16 |
| Visual | Visual | 0.88 | 36 | .385 | 0.29 |

*Note.* Independent samples t-tests revealed no mean differences pre- and post-upgrade for any network pair.

**Sustained Attention to Response Task**

The sustained attention to response task (SART) Fixed version (Manly et al. 2003) was conducted in E-prime at each wave. In each 1439ms trial, participants were successively presented with the digits 1-9 for 313ms, mask (circle with a cross inside) for 125ms, response cue (circle with bolded cross) for 63ms, mask again for 375ms and fixation cross for 563ms (see Figure S1). Participants were asked to respond as quickly and accurately as possible when the response cue appeared on the screen in every trial, except following presentation of the digit 3. A SART cycle consisted of nine trials, presenting digits 1 to 9 in ascending order. After each SART cycle, the next cycle recommenced at digit 1 immediately. Following detailed task instructions and a practice block of 13 trials, the full task consisted of 25 successive SART cycles (225 trials total), which lasted approximately 5.5 min.

***
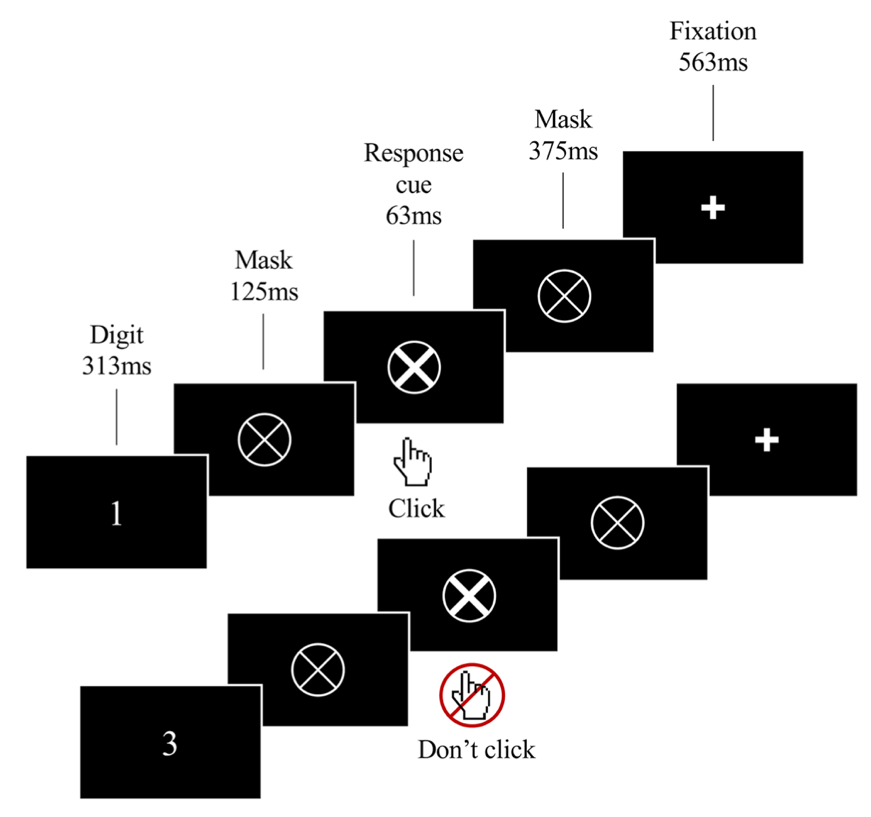
***

Figure S1. Procedure of the Sustained Attention to Response Task (Thomson et al., 2021). Adapted from Johnson, Robertson, et al. (2007).

**Attrition analyses**

Attrition analyses revealed that those attending only one wave had significantly lower IQ than those attending two (*p* = .020, *d* = - 0.64) or three (*p* = .006, *d* = -0.65) waves. There were no other significant differences between participants attending one, two or three waves in any demographic measures (range *p* = .052-.910). The number of participants who attended one, two or three waves was comparable between the ADHD and control groups (*p* = .514).

**Sample characteristics**

Table S2. Sample Characteristics (Sustained Attention Development)

|  | ADHD | Control | Test of significance |
| --- | --- | --- | --- |
| Total data points [% male] | 162 (75%) | 175 (57%) | - |
| Participants wave 1 [% male] | 51 (75%) | 58 (55%) | - |
| Participants wave 2 [% male] | 63 (75%) | 66 (56%) | - |
| Participants wave 3 [% male] | 48 (75%) | 51 (61%) | - |
| Age wave 1 [mean (SD)] | 10.4 (0.5) | 10.5 (0.5) | *t*(107) = -0.27, *p =* .784, *d =* -0.05 |
| Age wave 2 [mean (SD)] | 11.7 (0.6) | 11.8 (0.5) | *t*(127) = -0.79, *p =* .432, *d =* -0.14 |
| Age wave 3 [mean (SD)] | 13.3 (0.6) | 13.2 (0.5) | *t*(97) = 1.1, *p =* .281, *d =* 0.22 |
| % Male Participants | 77 (77%) | 82 (56%) | ***Χ*²(1,159) = 7.46, *p =* .006, *V =* 0.22** |
| IQ [mean (SD)] | 97 (14) | 104 (13) | ***t*(157) = -3.21, *p =* .002, *d =* -0.51** |
| SES [mean (SD)] | 1020 (39) | 1018 (47) | *t*(157) = 0.37, *p =* .712, *d =* 0.06 |
| DISC-IV Inattention symptoms [median (IQR)] | 7.0 (3.0) | 0.0 (2.0) | ***U =* 5898, *p <* .001, *r* = 0.87** |
| DISC-IV Hyperactivity-Impulsivity symptoms [median (IQR)] | 5.0 (3.5) | 0.0 (1.0) | ***U =* 5729, *p <* .001, *r* = 0.82** |
| Internalizing comorbidity [count (% group)] | 19 (25%) | 8 (10%) | ***Χ*²(1,158) = 6.10, *p =* .014, *V =* 0.20** |
| Externalizing comorbidity [count (% group)] | 39 (51%) | 8 (10%) | ***Χ*²(1,158) = 31.40, *p <* .001, *V =* 0.45** |
| Framewise displacement [median (IQR)] | 0.12 (0.11) | 0.10 (0.07) | ***U =* 16131, *p =* .006, *r* = 0.17** |

*Note.* Intelligence quotient (IQ) based on Wechsler Abbreviated Scale of Intelligence (Wechsler, 1999). Socioeconomic status (SES) based on SEIFA (Index of Relative Socio-Economic Advantage and Disadvantage; Australian Bureau of Statistics, 2013). ADHD symptom levels and presence of internalizing (depression, dysthymia, separation anxiety disorder, social phobia, generalized anxiety disorder, post-traumatic stress disorder, obsessive-compulsive disorder, hypomania or manic episode) or externalizing problems (oppositional defiant disorder or conduct disorder) based on the Diagnostic Interview Schedule for Children IV (DISC-IV) at baseline (Shaffer et al., 2000). Framewise displacement (in-scanner head motion; Jenkinson et al., 2002) also correlated with age across the sample, *r_s_* = -.14, *p* = .010. Bold indicates significant group difference. SD = standard deviation.

**Model fit statistics**

Table S3. Fit Statistics for Models of Network Functional Connectivity Developmen

| Connection of interest | | |  | |  | | | |  | | | Fit statistics | | | | | | | | | | | | |
| --- | --- | --- | --- | --- | --- | --- | --- | --- | --- | --- | --- | --- | --- | --- | --- | --- | --- | --- | --- | --- | --- | --- | --- | --- |
| Network 1 | Network 2 | | | | Model | | AIC | | | | BIC | | | | Log likelihood | | | Test | | | Likelihood ratio | | | *p*-value |
| Default Mode | | Default Mode | | | 1 | 2654.08 | | | | 2682.97 | | | -1322.04 | | | |  | | |  | | |  | |
| Default Mode | | Default Mode | | | 2 | 2641.40 | | | | 2676.07 | | | -1314.70 | | | | 1 vs. 2 | | | 14.67 | | | .000 | |
| Default Mode | | Default Mode | | | **3** | **2639.39** | | | | **2679.84** | | | **-1312.70** | | | | 2 vs. 3 | | | 4.01 | | | .045 | |
| Default Mode | | Dorsal Attention | | | 1 | 2889.81 | | | | 2920.14 | | | -1439.91 | | | |  | | |  | | |  | |
| Default Mode | | Dorsal Attention | | | 2 | 2882.65 | | | | 2919.05 | | | -1435.33 | | | | 1 vs. 2 | | | 9.16 | | | .003 | |
| Default Mode | | Dorsal Attention | | | **3** | **2870.22** | | | | **2912.68** | | | **-1428.11** | | | | 2 vs. 3 | | | 14.43 | | | < .001 | |
| Default Mode | | Frontoparietal | | | 1 | 3639.88 | | | | 3672.23 | | | -1814.94 | | | |  | | |  | | |  | |
| Default Mode | | Frontoparietal | | | 2 | 3606.01 | | | | 3644.84 | | | -1797.01 | | | | 1 vs. 2 | | | 35.86 | | | < .001 | |
| Default Mode | | Frontoparietal | | | **3** | **3588.53** | | | | **3633.83** | | | **-1787.26** | | | | 2 vs. 3 | | | 19.48 | | | < .001 | |
| Default Mode | | Limbic | | | 1 | 2673.09 | | | | 2703.42 | | | -1331.55 | | | |  | | |  | | |  | |
| Default Mode | | Limbic | | | **2** | **2633.52** | | | | **2669.92** | | | **-131.76** | | | | 1 vs. 2 | | | 41.57 | | | < .001 | |
| Default Mode | | Limbic | | | 3 | 2631.85 | | | | 2674.31 | | | -1308.93 | | | | 2 vs. 3 | | | 3.67 | | | .055 | |
| Default Mode | | Salience/Ventral Attention | | | 1 | 2015.70 | | | | 2046.03 | | | -1002.85 | | | |  | | |  | | |  | |
| Default Mode | | Salience/Ventral Attention | | | 2 | 1991.80 | | | | 2028.20 | | | -989.90 | | | | 1 vs. 2 | | | 25.89 | | | < .001 | |
| Default Mode | | Salience/Ventral Attention | | | **3** | **1983.25** | | | | **2025.71** | | | **-984.62** | | | | 2 vs. 3 | | | 10.56 | | | .001 | |
| Default Mode | | Somatomotor | | | 1 | 1815.86 | | | | 1846.19 | | | -902.93 | | | |  | | |  | | |  | |
| Default Mode | | Somatomotor | | | 2 | 1809.33 | | | | 1845.73 | | | -898.67 | | | | 1 vs. 2 | | | 8.53 | | | .004 | |
| Default Mode | | Somatomotor | | | **3** | **1803.39** | | | | **1845.85** | | | **-894.70** | | | | 2 vs. 3 | | | 7.94 | | | .005 | |
| Default Mode | | Visual | | | **1** | **3653.32** | | | | **3683.65** | | | **-1821.66** | | | |  | | |  | | |  | |
| Default Mode | | Visual | | | 2 | 3653.94 | | | | 3690.33 | | | -1820.97 | | | | 1 vs. 2 | | | 1.39 | | | .239 | |
| Dorsal Attention | | Dorsal Attention | | | **1** | **261.06** | | | | **280.99** | | | **-125.53** | | | |  | | |  | | |  | |
| Dorsal Attention | | Dorsal Attention | | | 2 | 263.03 | | | | 286.95 | | | -125.51 | | | | 1 vs. 2 | | | 0.03 | | | .861 | |
| Dorsal Attention | | Frontoparietal | | | 1 | 1280.71 | | | | 1309.60 | | | -635.35 | | | |  | | |  | | |  | |
| Dorsal Attention | | Frontoparietal | | | **2** | **1261.94** | | | | **1296.61** | | | **-624.97** | | | | 1 vs. 2 | | | 20.76 | | | < .001 | |
| Dorsal Attention | | Frontoparietal | | | 3 | 1262.51 | | | | 1302.96 | | | -624.25 | | | | 2 vs. 3 | | | 1.43 | | | .231 | |
| Dorsal Attention | | Limbic | | | 1 | 1257.55 | | | | 1284.41 | | | -623.77 | | | |  | | |  | | |  | |
| Dorsal Attention | | Limbic | | | 2 | 1247.35 | | | | 1279.58 | | | -617.67 | | | | 1 vs. 2 | | | 12.2 | | | .001 | |
| Dorsal Attention | | Limbic | | | **3** | **1233.61** | | | | **1271.22** | | | **-609.81** | | | | 2 vs. 3 | | | 15.74 | | | < .001 | |
| Dorsal Attention | | Salience/Ventral Attention | | | 1 | 1544.71 | | | | 1571.58 | | | -767.36 | | | |  | | |  | | |  | |
| Dorsal Attention | | Salience/Ventral Attention | | | 2 | 1536.77 | | | | 1569.00 | | | -762.38 | | | | 1 vs. 2 | | | 9.95 | | | .002 | |
| Dorsal Attention | | Salience/Ventral Attention | | | **3** | **1534.18** | | | | **1571.79** | | | **-760.09** | | | | 2 vs. 3 | | | 4.58 | | | .032 | |
| Dorsal Attention | | Somatomotor | | | 1 | 603.86 | | | | 630.72 | | | -296.93 | | | |  | | |  | | |  | |
| Dorsal Attention | | Somatomotor | | | **2** | **600.34** | | | | **632.57** | | | **-294.17** | | | | 1 vs. 2 | | | 5.52 | | | .019 | |
| Dorsal Attention | | Somatomotor | | | 3 | 602.29 | | | | 639.90 | | | -294.15 | | | | 2 vs. 3 | | | 0.04 | | | .839 | |
| Dorsal Attention | | Visual | | | **1** | **1839.78** | | | | **1866.64** | | | **-914.89** | | | |  | | |  | | |  | |
| Dorsal Attention | | Visual | | | 2 | 1839.65 | | | | 1871.88 | | | -913.82 | | | | 1 vs. 2 | | | 2.13 | | | .144 | |
| Frontoparietal | | Frontoparietal | | | 1 | 1165.95 | | | | 1191.38 | | | -577.98 | | | |  | | |  | | |  | |
| Frontoparietal | | Frontoparietal | | | **2** | **1158.27** | | | | **1188.78** | | | **-573.14** | | | | 1 vs. 2 | | | 9.68 | | | .002 | |
| Frontoparietal | | Frontoparietal | | | 3 | 1156.65 | | | | 1192.25 | | | -571.33 | | | | 2 vs. 3 | | | 3.62 | | | .057 | |
| Frontoparietal | | Limbic | | | 1 | 2012.78 | | | | 2041.67 | | | -1001.39 | | | |  | | |  | | |  | |
| Frontoparietal | | Limbic | | | 2 | 1979.62 | | | | 2014.29 | | | -983.81 | | | | 1 vs. 2 | | | 35.16 | | | < .001 | |
| Frontoparietal | | Limbic | | | **3** | **1975.52** | | | | **2015.96** | | | **-980.76** | | | | 2 vs. 3 | | | 6.1 | | | .014 | |
| Frontoparietal | | Salience/Ventral Attention | | | 1 | 1945.22 | | | | 1974.11 | | | -967.61 | | | |  | | |  | | |  | |
| Frontoparietal | | Salience/Ventral Attention | | | 2 | 1930.36 | | | | 1965.03 | | | -959.18 | | | | 1 vs. 2 | | | 16.86 | | | < .001 | |
| Frontoparietal | | Salience/Ventral Attention | | | **3** | **1920.95** | | | | **1961.39** | | | **-953.47** | | | | 2 vs. 3 | | | 11.42 | | | .001 | |
| Frontoparietal | | Somatomotor | |  | **1** | | | **1078.92** | | **1107.81** | | | | **-534.46** | |  | | |  | | |  | | |
| Frontoparietal | | Somatomotor | |  | 2 | | | 1078.11 | | 1112.78 | | | | -533.05 | | 1 vs. 2 | | | 2.81 | | | .094 | | |
| Frontoparietal | | Visual | |  | 1 | | | 1142.89 | | 1171.78 | | | | -566.44 | |  | | |  | | |  | | |
| Frontoparietal | | Visual | |  | 2 | | | 1130.71 | | 1165.38 | | | | -559.36 | | 1 vs. 2 | | | 14.17 | | | < .001 | | |
| Frontoparietal | | Visual | |  | **3** | | | **1112.59** | | **1153.03** | | | | **-549.29** | | 2 vs. 3 | | | 20.13 | | | < .001 | | |
| Limbic | | Limbic | |  | **1** | | | **213.09** | | **233.03** | | | | **-101.55** | |  | | |  | | |  | | |
| Limbic | | Limbic | |  | 2 | | | 215.03 | | 238.95 | | | | -101.52 | | 1 vs. 2 | | | 0.06 | | | .806 | | |
| Limbic | | Salience/Ventral Attention | | | 1 | | | 670.44 | | 697.30 | | | | -330.22 | |  | | |  | | |  | | |
| Limbic | | Salience/Ventral Attention | | | 2 | | | 655.86 | | 688.10 | | | | -321.93 | | 1 vs. 2 | | | 16.57 | | | < .001 | | |
| Limbic | | Salience/Ventral Attention | | | **3** | | | **652.23** | | **689.84** | | | | **-319.11** | | 2 vs. 3 | | | 5.64 | | | .018 | | |
| Limbic | | Somatomotor | |  | 1 | | | 1128.98 | | 1155.85 | | | | -559.49 | |  | | |  | | |  | | |
| Limbic | | Somatomotor | |  | **2** | | | **1121.24** | | **1153.48** | | | | **-554.62** | | 1 vs. 2 | | | 9.74 | | | .002 | | |
| Limbic | | Somatomotor | |  | 3 | | | 1123.23 | | 1160.84 | | | | -554.61 | | 2 vs. 3 | | | 0.02 | | | .897 | | |
| Limbic | | Visual | |  | 1 | | | 593.90 | | 62.76 | | | | -291.95 | |  | | |  | | |  | | |
| Limbic | | Visual | |  | 2 | | | 589.05 | | 621.29 | | | | -288.53 | | 1 vs. 2 | | | 6.84 | | | .009 | | |
| Limbic | | Visual | |  | **3** | | | **578.69** | | **616.30** | | | | **-282.34** | | 2 vs. 3 | | | 12.37 | | | < .001 | | |
| Salience/Ventral Attention | | Salience/Ventral Attention | | | **1** | | | **316.11** | | **336.04** | | | | **-153.05** | |  | | |  | | |  | | |
| Salience/Ventral Attention | | Salience/Ventral Attention | | | 2 | | | 317.69 | | 341.60 | | | | -152.84 | | 1 vs. 2 | | | 0.42 | | | .516 | | |
| Salience/Ventral Attention | | Somatomotor | |  | 1 | | | 807.65 | | 834.52 | | | | -398.83 | |  | | |  | | |  | | |
| Salience/Ventral Attention | | Somatomotor | |  | 2 | | | 800.70 | | 832.94 | | | | -394.35 | | 1 vs. 2 | | | 8.95 | | | .003 | | |
| Salience/Ventral Attention | | Somatomotor | |  | **3** | | | **789.63** | | **827.24** | | | | **-387.81** | | 2 vs. 3 | | | 13.07 | | | < .001 | | |
| Salience/Ventral Attention | | Visual | |  | 1 | | | 1126.77 | | 1153.63 | | | | -558.39 | |  | | |  | | |  | | |
| Salience/Ventral Attention | | Visual | |  | 2 | | | 1123.26 | | 1155.49 | | | | -555.63 | | 1 vs. 2 | | | 5.51 | | | .019 | | |
| Salience/Ventral Attention | | Visual | |  | **3** | | | **1112.30** | | **1149.91** | | | | **-549.15** | | 2 vs. 3 | | | 12.95 | | | < .001 | | |
| Somatomotor | | Somatomotor | |  | **1** | | | **196.01** | | **215.95** | | | | **-93.01** | |  | | |  | | |  | | |
| Somatomotor | | Somatomotor | |  | 2 | | | 197.71 | | 221.63 | | | | -92.86 | | 1 vs. 2 | | | 0.30 | | | .583 | | |
| Somatomotor | | Visual | |  | **1** | | | **1232.12** | | **1258.98** | | | | **-611.06** | |  | | |  | | |  | | |
| Somatomotor | | Visual | |  | 2 | | | 1231.28 | | 1263.52 | | | | -609.64 | | 1 vs. 2 | | | 2.83 | | | .092 | | |
| Visual | | Visual | |  | **1** | | | **95.55** | | **115.48** | | | | **-42.78** | |  | | |  | | |  | | |
| Visual | | Visual | |  | 2 | | | 97.47 | | 121.39 | | | | -42.74 | | 1 vs. 2 | | | 2.83 | | | .092 | | |

*Note.* Bold indicates selected model. AIC = Akaike Information Criterion. BIC = Bayesian Information Criterion.

Table S4. Fit Statistics for Models of Network Sustained Attention Development

| Connection of interest | | | Fit statistics | | | | | | |  |  |
| --- | --- | --- | --- | --- | --- | --- | --- | --- | --- | --- | --- |
|  |  |  |  |  |  |  |  |  |  |  |  |
| Network 1 | Network 2 | Model | | AIC | BIC | Log likelihood | Test | Likelihood ratio | *p*-value |  | |
| Default Mode | Frontoparietal | 1 | | 36786.42 | 36836.86 | -18385.21 |  |  |  |  |  |
|  |  | 2 | | 36773.39 | 36830.14 | -18377.69 | 1 vs. 2 | 15.03 | <.001 |  |  |
|  |  | **3** | | **36766.33** | **36829.38** | **-18373.16** | 2 vs. 3 | 9.06 | .003 |  |  |
|  |  | 4 | | 36765.41 | 36847.37 | -18369.70 | 3 vs. 4 | 6.92 | .075 |  |  |
| Default Mode | Limbic | 1 | | 24993.38 | 25040.58 | -12488.69 |  |  |  |  |  |
|  |  | 2 | | 24988.99 | 25042.08 | -12485.49 | 1 vs. 2 | 6.39 | .011 |  |  |
|  |  | **3** | | **24982.38** | **25041.38** | **-12481.19** | 2 vs. 3 | 8.60 | .003 |  |  |
|  |  | 4 | | 24984.01 | 25060.70 | -12479.00 | 3 vs. 4 | 4.38 | .223 |  |  |
| Default Mode | Salience/Ventral Attention | 1 | | 24993.38 | 25040.58 | -12488.69 |  |  |  |  |  |
|  |  | 2 | | 24990.86 | 25043.95 | -12486.43 | 1 vs. 2 | 4.53 | .033 |  |  |
|  |  | **3** | | **24980.88** | **25039.88** | **-12480.44** | 2 vs. 3 | 11.97 | .001 |  |  |
|  |  | 4 | | 24981.13 | 25057.83 | -12477.57 | 3 vs. 4 | 5.75 | .124 |  |  |
| Dorsal Attention | Frontoparietal | 1 | | 19070.03 | 19114.92 | -9527.02 |  |  |  |  |  |
|  |  | 2 | | 19063.45 | 19113.96 | -9522.73 | 1 vs. 2 | 8.58 | .003 |  |  |
|  |  | 3 | | 19044.23 | 19100.35 | -9512.11 | 2 vs. 3 | 21.22 | <.001 |  |  |
|  |  | **4** | | **19040.85** | **19113.80** | **-9507.42** | 3 vs. 4 | 9.38 | .025 |  |  |
| Dorsal Attention | Limbic | 1 | | 13107.12 | 13148.77 | -6545.56 |  |  |  |  |  |
|  |  | 2 | | 13103.48 | 13150.33 | -6542.74 | 1 vs. 2 | 5.64 | .018 |  |  |
|  |  | **3** | | **13100.87** | **13152.93** | **-6540.44** | 2 vs. 3 | 4.61 | .032 |  |  |
|  |  | 4 | | 13103.86 | 13171.54 | -6538.93 | 3 vs. 4 | 3.01 | .390 |  |  |
| Frontoparietal | Frontoparietal | 1 | | 10094.61 | 10133.96 | -5039.31 |  |  |  |  |  |
|  |  | 2 | | 10089.41 | 10133.68 | -5035.71 | 1 vs. 2 | 7.20 | .007 |  |  |
|  |  | **3** | | **10084.75** | **10133.94** | **-5032.38** | 2 vs. 3 | 6.66 | .010 |  |  |
|  |  | 4 | | 10090.06 | 10154.00 | -5032.03 | 3 vs. 4 | 0.69 | .875 |  |  |
| Frontoparietal | Limbic | 1 | | 19070.03 | 19114.92 | -9527.02 |  |  |  |  |  |
|  |  | 2 | | 19065.73 | 19116.24 | -9523.87 | 1 vs. 2 | 6.30 | .012 |  |  |
|  |  | **3** | | **19056.19** | **19112.31** | **-9518.09** | 2 vs. 3 | 11.54 | .001 |  |  |
|  |  | 4 | | 19058.57 | 19131.52 | -9516.28 | 3 vs. 4 | 3.62 | .305 |  |  |
| Frontoparietal | Salience/Ventral Attention | 1 | | 19070.03 | 19114.92 | -9527.02 |  |  |  |  |  |
|  |  | 2 | | 19067.08 | 19117.59 | -9524.54 | 1 vs. 2 | 4.95 | .026 |  |  |
|  |  | **3** | | **19060.06** | **19116.18** | **-9520.03** | 2 vs. 3 | 9.02 | .003 |  |  |
|  |  | 4 | | 19062.88 | 19135.83 | -9518.44 | 3 vs. 4 | 3.18 | .364 |  |  |
| Frontoparietal | Somatomotor | 1 | | 19070.03 | 19114.92 | -9527.02 |  |  |  |  |  |
|  |  | 2 | | 19067.11 | 19117.62 | -9524.56 | 1 vs. 2 | 4.92 | .027 |  |  |
|  |  | **3** | | **19061.17** | **19117.28** | **-9520.58** | 2 vs. 3 | 7.95 | .005 |  |  |
|  |  | 4 | | 19063.56 | 19136.51 | -9518.78 | 3 vs. 4 | 3.61 | .307 |  |  |
| Frontoparietal | Visual | 1 | | 19070.03 | 19114.92 | -9527.02 |  |  |  |  |  |
|  |  | 2 | | 19068.03 | 19118.54 | -9525.02 | 1 vs. 2 | 4.00 | .046 |  |  |
|  |  | **3** | | **19060.34** | **19116.46** | **-9520.17** | 2 vs. 3 | 9.69 | .002 |  |  |
|  |  | 4 | | 19061.04 | 19133.99 | -9517.52 | 3 vs. 4 | 5.30 | .151 |  |  |
| Salience/Ventral Attention | Somatomotor | 1 | | 13107.12 | 13148.77 | -6545.56 |  |  |  |  |  |
|  |  | 2 | | 13094.55 | 13141.40 | -6538.27 | 1 vs. 2 | 14.57 | <.001 |  |  |
|  |  | **3** | | **13079.84** | **13131.91** | **-6529.92** | 2 vs. 3 | 16.71 | <.001 |  |  |
|  |  | 4 | | 13080.39 | 13148.07 | -6527.20 | 3 vs. 4 | 5.45 | .142 |  |  |

*Note.* Bold indicates selected model. AIC = Akaike Information Criterion. BIC = Bayesian Information Criterion.

**Model parameters**

Table S5. Linear Mixed Effects Models of Network Functional Connectivity Development

| Connection of interest | | Variable [Regression Coefficient (standard error), FDR-corrected p-value] | | | | |
| --- | --- | --- | --- | --- | --- | --- |
| Network 1 | Network 2 | (Intercept) | Age | Sex | Group | Age x Group |
| Default Mode | Default Mode | **0.76 (0.04), *p* < .001** | -0.04 (0.02), *p* = .092* | 0.04 (0.04), *p* = .538 | -0.03 (0.04), *p* = .642 | 0.03 (0.03), *p* = .394 |
| Default Mode | Dorsal Attention | **0.51 (0.04), *p* < .001** | -0.03 (0.02), *p* = .125 | 0.07 (0.04), *p* = .155 | -0.01 (0.04), *p* = .905 | 0.04 (0.02), *p* = .188 |
| Default Mode | Frontoparietal | **0.54 (0.04), *p* < .001** | -0.04 (0.02), *p* = .056* | 0.07 (0.04), *p* = .146 | -0.01 (0.04), *p* = .806 | 0.04 (0.02), *p* = .209 |
| Default Mode | Limbic | **0.36 (0.04), *p* < .001** | **-0.03 (0.01), *p* = .021** | **0.11 (0.04), *p* = .034** | -0.03 (0.04), *p* = .581 | - |
| Default Mode | Salience/Ventral Attention | **0.52 (0.04), *p* < .001** | -0.04 (0.02), *p* = .126 | 0.03 (0.04), *p* = .647 | -0.02 (0.04), *p* = .702 | 0.03 (0.03), *p* = .379 |
| Default Mode | Somatomotor | **0.53 (0.03), *p* < .001** | -0.02 (0.02), *p* = .425 | 0.03 (0.03), *p* = .429 | -0.01 (0.03), *p* = .883 | 0.02 (0.02), *p* = .637 |
| Default Mode | Visual | **0.68 (0.03), *p* < .001** | - | **0.09 (0.03), *p* = .021** | 0.02 (0.03), *p* = .687 | - |
| Dorsal Attention | Dorsal Attention | **0.51 (0.05), *p* < .001** | - | 0.06 (0.04), *p* = .307 | 0.02 (0.04), *p* = .758 | - |
| Dorsal Attention | Frontoparietal | **0.62 (0.03), *p* < .001** | -0.02 (0.01), *p* = .154 | 0.02 (0.03), *p* = .744 | -0.02 (0.03), *p* = .690 | - |
| Dorsal Attention | Limbic | **0.71 (0.05), *p* < .001** | -0.04 (0.02), *p* = .098* | 0.09 (0.05), *p* = .126 | -0.02 (0.04), *p* = .752 | 0.05 (0.03), *p* = .164 |
| Dorsal Attention | Salience/Ventral Attention | **0.83 (0.04), *p* < .001** | -0.04 (0.02), *p* = .129 | 0.05 (0.04), *p* = .301 | 0.00 (0.04), *p* = .945 | 0.03 (0.03), *p* = .332 |
| Dorsal Attention | Somatomotor | **0.52 (0.03), *p* < .001** | -0.01 (0.01), *p* = .348 | 0.07 (0.03), *p* = .081* | 0.00 (0.03), *p* = .938 | - |
| Dorsal Attention | Visual | **0.57 (0.04), *p* < .001** | - | **0.09 (0.04), *p* = .041** | 0.03 (0.03), *p* = .570 | - |
| Frontoparietal | Frontoparietal | **0.42 (0.04), *p* < .001** | -0.03 (0.01), *p* = .068* | 0.07 (0.04), *p* = .203 | -0.03 (0.04), *p* = .560 | - |
| Frontoparietal | Limbic | **0.48 (0.04), *p* < .001** | **-0.05 (0.02), *p* = .008** | 0.06 (0.04), *p* = .230 | -0.04 (0.04), *p* = .507 | 0.03 (0.02), *p* = .277 |
| Frontoparietal | Salience/Ventral Attention | **0.61 (0.04), *p* < .001** | **-0.04 (0.02), *p* = .024** | 0.05 (0.04), *p* = .309 | -0.01 (0.03), *p* = .799 | 0.04 (0.02), *p* = .150 |
| Frontoparietal | Somatomotor | **0.49 (0.03), *p* < .001** | - | 0.05 (0.03), *p* = .232 | -0.03 (0.03), *p* = .425 | - |
| Frontoparietal | Visual | **0.55 (0.04), *p* < .001** | **-0.04 (0.02), *p* = .042** | 0.08 (0.04), *p* = .068* | 0.02 (0.04), *p* = .740 | 0.05 (0.02), *p* = .097* |
| Limbic | Limbic | **0.56 (0.04), *p* < .001** | - | 0.01 (0.04), *p* = .812 | 0.01 (0.03), *p* = .804 | - |
| Limbic | Salience/Ventral Attention | **0.54 (0.04), *p* < .001** | -0.03 (0.02), *p* = .161 | 0.04 (0.04), *p* = .421 | -0.02 (0.04), *p* = .693 | 0.03 (0.02), *p* = .408 |
| Limbic | Somatomotor | **0.51 (0.04), *p* < .001** | -0.02 (0.01), *p* = .100* | 0.05 (0.04), *p* = .301 | -0.01 (0.04), *p* = .834 | - |
| Limbic | Visual | **0.56 (0.03), *p* < .001** | -0.03 (0.01), *p* = .058* | 0.07 (0.03), *p* = .097* | 0.01 (0.03), *p* = .795 | 0.04 (0.02), *p* = .106* |
| Salience/Ventral Attention | Salience/Ventral Attention | **0.78 (0.05), *p* < .001** | - | 0.06 (0.05), *p* = .376 | -0.03 (0.05), *p* = .626 | - |
| Salience/Ventral Attention | Somatomotor | **0.41 (0.04), *p* < .001** | -0.03 (0.02), *p* = .130 | 0.08 (0.04), *p* = .103* | -0.01 (0.04), *p* = .805 | 0.04 (0.03), *p* = .199 |
| Salience/Ventral Attention | Visual | **0.80 (0.03), *p* < .001** | **-0.04 (0.01), *p* = .017** | **0.08 (0.03), *p* = .049** | 0.01 (0.03), *p* = .811 | **0.05 (0.02), *p* = .047** |
| Somatomotor | Somatomotor | **0.49 (0.04), *p* < .001** | - | 0.06 (0.04), *p* = .203 | 0.02 (0.04), *p* = .700 | - |
| Somatomotor | Visual | **0.52 (0.04), *p* < .001** | - | 0.07 (0.03), *p* = .098 | 0.01 (0.03), *p* = .803 | - |
| Visual | Visual | **0.98 (0.03), *p* < .001** | - | 0.02 (0.03), *p* = .746 | -0.01 (0.03), *p* = .810 | - |

*Note*. All p-values are FDR corrected by the Benjamini-Hochberg method (Benjamini & Hochberg, 1995). All models include a random intercept; models with age variable include a random age slope. Bold identifies false discovery rate (FDR) corrected p-values < .05. * *p* < .05 prior to FDR-correction only. Hyphen indicates variable was not involved in best fitting model.

| Table S6. Linear Mixed Effects Models of Sustained Attention Development | | | | | | | |  |  |  |  |
| --- | --- | --- | --- | --- | --- | --- | --- | --- | --- | --- | --- |
| Connection of interest | | | Variable [Regression Coefficient (standard error), FDR-corrected p-value] | | | | | | | | |
| Network 1 | Network 2 | (Intercept) | | Age | Sex | Group | FC | | FC x Age | FC x Age x Group |  |
| Default Mode | Frontoparietal | **140.13 (10.85), *p <* .001** | | **-12.61 (3.59), *p =* .001** | -0.58 (10.68), *p =* 1.000 | **-23.51 (10.14), *p =* .032** | **3.58 (1.01), *p =* .001** | | **2.54 (0.84), *p =* .006** | - | |
| Default Mode | Limbic | **141.18 (10.83), *p <* .001** | | **-12.38 (3.57), *p =* .002** | -0.81 (10.66), *p =* 1.000 | **-23.46 (10.12), *p =* .032** | **2.76 (1.23), *p =* .033** | | **3.10 (1.06), *p =* .007** | - | |
| Default Mode | Salience/Ventral Attention | **140.94 (10.85), *p <* .001** | | **-13.37 (3.60), *p =* .001** | -0.47 (10.66), *p =* 1.000 | **-23.51 (10.12), *p =* .032** | 2.14 (1.46), *p =* .180 | | **4.09 (1.18), *p =* .002** | - | |
| Dorsal Attention | Frontoparietal | **136.53 (10.97), *p <* .001** | | **-14.99 (5.30), *p =* .010** | -0.04 (10.66), *p =* .997 | -18.48 (10.47), *p =* .103 | **7.67 (2.51), *p =* .005** | | **10.53 (2.12), *p <* .001** | **-7.10 (3.13), *p =* .033** | |
| Dorsal Attention | Limbic | **138.77 (10.88), *p <* .001** | | **-14.22 (3.77), *p =* .001** | -0.87 (10.60), *p =* 1.000 | **-23.7 (10.07), *p =* .035** | **4.74 (2.20), *p =* .042** | | **3.95 (1.83), *p =* .043** | - | |
| Frontoparietal | Frontoparietal | **140.05 (10.73), *p <* .001** | | **-13.37 (3.56), *p =* .001** | -1.28 (10.53), *p =* 1.000 | **-23.89 (10.00), *p =* .033** | **6.07 (2.35), *p =* .018** | | **5.20 (2.01), *p =* .018** | - | |
| Frontoparietal | Limbic | **140.62 (10.82), *p <* .001** | | **-13.20 (3.58), *p =* .001** | -0.77 (10.63), *p =* 1.000 | **-23.58 (10.1), *p =* .032** | **3.45 (1.48), *p =* .031** | | **4.29 (1.26), *p =* .002** | - | |
| Frontoparietal | Salience/Ventral Attention | **140.41 (10.83), *p <* .001** | | **-13.47 (3.61), *p =* .001** | -0.52 (10.63), *p =* 1.000 | **-23.61 (10.09), *p =* .032** | 2.75 (1.48), *p =* .082* | | **3.60 (1.20), *p =* .006** | - | |
| Frontoparietal | Somatomotor | **140.13 (10.83), *p <* .001** | | **-13.59 (3.62), *p =* .001** | -0.57 (10.63), *p =* 1.000 | **-23.38 (10.09), *p =* .031** | 3.81 (1.98), *p =* .073* | | **4.68 (1.66), *p =* .010** | - | |
| Frontoparietal | Visual | **140.11 (10.86), *p <* .001** | | **-14.15 (3.66), *p <* .001** | -0.65 (10.65), *p =* 1.000 | **-23.79 (10.12), *p =* .035** | 3.48 (1.85), *p =* .079* | | **4.77 (1.53), *p =* .004** | - | |
| Salience/Ventral Attention | Somatomotor | **138.75 (10.75), *p <* .001** | | **-15.22 (3.64), *p <* .001** | -1.08 (10.53), *p =* 1.000 | **-23.72 (10.00), *p =* .034** | **8.43 (2.58), *p =* .003** | | **8.65 (2.11), *p <* .001** | - | |

*Note*. All p-values are FDR corrected by the Benjamini-Hochberg method (Benjamini & Hochberg, 1995). All models include a random intercept and random age slope. Bold identifies false discovery rate (FDR) corrected p-values < .05. * *p* < .05 prior to FDR-correction only. Model including dorsal attention to frontoparietal functional connectivity (FC) also contained lower order interactions: FC x Group, -6.40 (3.73), *p* = .111; and Age x Group, -0.89 (7.35), *p* = 1.000. Hyphen indicates variable was not involved in best fitting model.

**Models with ADHD medication covariate**

Given a small number of participants were taking ADHD medication on the assessment day, models were rerun with the inclusion of ADHD medication use as a covariate (see Tables S7-8). Results are largely unchanged with the inclusion of ADHD medication use and medication use was not significant in any model.

Table S7. Linear Mixed Effects Models of Functional Connectivity Development (covarying for ADHD medication use)

| Connection of interest | |  | Variable [Regression Coefficient (standard error), FDR-corrected p-value] | | | | | | |
| --- | --- | --- | --- | --- | --- | --- | --- | --- | --- |
| Network 1 | Network 2 | Model form | (Intercept) | Age | Sex | Group | Age x Group | ADHD Medication |  |
| Default Mode | Default Mode | A3 | **0.77 (0.04)***** | -0.04 (0.02)^ | 0.04 (0.04) | -0.04 (0.04) | 0.03 (0.03) | -0.03 (0.06) |  |
| Default Mode | Dorsal Attention | A3 | **0.51 (0.04)***** | -0.03 (0.02)^ | 0.07 (0.04) | -0.01 (0.04) | 0.04 (0.02) | -0.03 (0.05) |  |
| Default Mode | Frontoparietal | A3 | **0.54 (0.04)***** | -0.04 (0.02)^ | 0.07 (0.04) | -0.02 (0.04) | 0.04 (0.02) | -0.01 (0.05) |  |
| Default Mode | Limbic | A2 | **0.36 (0.04)***** | **-0.03 (0.01)*** | **0.11 (0.04)*** | -0.04 (0.04) | - | -0.03 (0.05) |  |
| Default Mode | Salience/Ventral Attention | A3 | **0.52 (0.04)***** | -0.04 (0.02) | 0.03 (0.04) | -0.02 (0.04) | 0.03 (0.03) | -0.01 (0.05) |  |
| Default Mode | Somatomotor | A3 | **0.53 (0.03)***** | -0.02 (0.02) | 0.03 (0.03) | -0.01 (0.03) | 0.02 (0.02) | -0.03 (0.04) |  |
| Default Mode | Visual | A1 | **0.68 (0.03)***** | - | **0.09 (0.03)*** | 0.01 (0.03) | - | -0.02 (0.04) |  |
| Dorsal Attention | Dorsal Attention | A1 | **0.52 (0.05)***** | - | 0.05 (0.05) | 0.00 (0.05) | - | -0.04 (0.06) |  |
| Dorsal Attention | Frontoparietal | A2 | **0.62 (0.04)***** | -0.02 (0.01) | 0.02 (0.03) | -0.02 (0.03) | - | -0.02 (0.05) |  |
| Dorsal Attention | Limbic | A3 | **0.73 (0.05)***** | -0.04 (0.02)^ | 0.09 (0.05)^ | -0.04 (0.05) | 0.05 (0.03) | -0.08 (0.06) |  |
| Dorsal Attention | Salience/Ventral Attention | A3 | **0.84 (0.04)***** | -0.04 (0.02) | 0.06 (0.04) | -0.01 (0.04) | 0.03 (0.03) | -0.03 (0.06) |  |
| Dorsal Attention | Somatomotor | A2 | **0.53 (0.03)***** | -0.01 (0.01) | 0.07 (0.03)^ | -0.01 (0.03) | - | -0.04 (0.05) |  |
| Dorsal Attention | Visual | A1 | **0.58 (0.04)***** | - | **0.09 (0.04)*** | 0.02 (0.04) | - | -0.04 (0.05) |  |
| Frontoparietal | Frontoparietal | A2 | **0.42 (0.05)***** | -0.03 (0.01)^ | 0.07 (0.04) | -0.04 (0.04) | - | 0.00 (0.06) |  |
| Frontoparietal | Limbic | A3 | **0.48 (0.04)***** | **-0.05 (0.02)*** | 0.06 (0.04) | -0.04 (0.04) | 0.03 (0.02) | 0.00 (0.06) |  |
| Frontoparietal | Salience/Ventral Attention | A3 | **0.61 (0.04)***** | **-0.04 (0.02)*** | 0.05 (0.04) | -0.02 (0.04) | 0.04 (0.02) | -0.02 (0.05) |  |
| Frontoparietal | Somatomotor | A1 | **0.49 (0.03)***** | - | 0.05 (0.03) | -0.04 (0.03) | - | -0.03 (0.04) |  |
| Frontoparietal | Visual | A3 | **0.55 (0.04)***** | **-0.04 (0.02)*** | 0.09 (0.04)^ | 0.01 (0.04) | 0.05 (0.02)^ | -0.02 (0.05) |  |
| Limbic | Limbic | A1 | **0.58 (0.04)***** | - | 0.01 (0.04) | -0.01 (0.04) | - | -0.08 (0.05) |  |
| Limbic | Salience/Ventral Attention | A3 | **0.54 (0.04)***** | -0.03 (0.02) | 0.04 (0.04) | -0.02 (0.04) | 0.03 (0.02) | -0.01 (0.05) |  |
| Limbic | Somatomotor | A2 | **0.52 (0.04)***** | -0.02 (0.01)^ | 0.05 (0.04) | -0.02 (0.04) | - | -0.03 (0.05) |  |
| Limbic | Visual | A3 | **0.57 (0.03)***** | -0.03 (0.01)^ | 0.07 (0.03)^ | 0.00 (0.03) | 0.04 (0.02)^ | -0.05 (0.05) |  |
| Salience/Ventral Attention | Salience/Ventral Attention | A1 | **0.79 (0.05)***** | - | 0.06 (0.05) | -0.04 (0.05) | - | -0.02 (0.07) |  |
| Salience/Ventral Attention | Somatomotor | A3 | **0.41 (0.04)***** | -0.03 (0.02) | 0.08 (0.04)^ | -0.02 (0.04) | 0.04 (0.03) | -0.01 (0.05) |  |
| Salience/Ventral Attention | Visual | A3 | **0.81 (0.04)***** | **-0.04 (0.01)*** | 0.08 (0.03)^ | 0.00 (0.03) | 0.05 (0.02)^ | -0.04 (0.05) |  |
| Somatomotor | Somatomotor | A1 | **0.50 (0.04)***** | - | 0.06 (0.04) | 0.01 (0.04) | - | -0.03 (0.06) |  |
| Somatomotor | Visual | A1 | **0.53 (0.04)***** | - | 0.07 (0.04)^ | 0.00 (0.04) | - | -0.04 (0.05) |  |
| Visual | Visual | A1 | **1.00 (0.04)***** | - | 0.02 (0.03) | -0.03 (0.03) | - | -0.08 (0.05) |  |

*Note* Bold identifies significant independent variable following false discovery rate (FDR) correction from the Benjamini-Hochberg method (Benjamini & Hochberg, 1995). See equations for final models A1-3 in methods. All models include a random intercept; models with age variable include a random age slope. ADHD medication use represents dichotomous (yes/no) variable if any of methylphenidate, clonidine, atomoxetine or lisdexamfetamine were taken on an assessment day. * FDR-corrected *p* < .050, ** FDR-corrected *p* < .010, *** FDR-corrected *p* < .001, ^ *p* < .050 prior to FDR-correction only. Hyphen indicates variable was not involved in best fitting model.

| Table S8. Linear Mixed Effects Models of Sustained Attention Development (covarying for ADHD medication use) | | | | | | | | | | | | | | |  |
| --- | --- | --- | --- | --- | --- | --- | --- | --- | --- | --- | --- | --- | --- | --- | --- |
| Connection of interest | |  | | Variable [Regression Coefficient (standard error), FDR-corrected p-value] | | | | | | | | | |  |  |
| Network 1 | Network 2 | Model form | (Intercept) | | Age | Sex | Group | FC | FC x Age | FC x Age x Group | ADHD Medication |  | |  |  |
| Default Mode | Frontoparietal | B3 | **142.60 (11.29)***** | | **-13.15 (3.60)***** | -0.05 (10.72) | **-24.97 (10.73)*** | 1.20  (1.18) | **3.50 (0.99)**** | - | -6.83  (16.36) | |  | | |
| Default Mode | Limbic | B3 | **142.38 (11.28)***** | | **-12.39 (3.58)**** | -0.48 (10.72) | **-24.83 (10.73)*** | **2.76 (1.23)*** | **3.10 (1.06)**** | - | -6.50  (16.34) | |  | | |
| Default Mode | Salience/ventral attention | B3 | **142.18 (11.29)***** | | **-13.38 (3.60)***** | -0.14 (10.71) | **-24.92 (10.72)*** | 2.13  (1.46) | **4.09 (1.18)**** | - | -6.75  (16.34) | |  | | |
| Dorsal Attention | Frontoparietal | B4 | **137.76 (11.42)***** | | **-15.02 (5.30)*** | 0.28 (10.71) | -19.88 (11.06) | **7.65 (2.52)**** | **10.54 (2.12)***** | **-7.11 (3.13)*** | -6.56  (16.35) | |  | | |
| Dorsal Attention | Limbic | B3 | **139.89 (11.34)***** | | **-14.23 (3.77)***** | -0.58 (10.66) | **-24.96 (10.67)*** | 4.72  (2.20)^ | 3.95 (1.84)^ | - | -5.98  (16.29) | |  | | |
| Frontoparietal | Frontoparietal | B3 | **141.31 (11.18)***** | | **-13.38 (3.57)***** | -0.97 (10.58) | **-25.31 (10.59)*** | **6.05 (2.35)*** | **5.21 (2.01)*** | - | -6.73  (16.18) | |  | | |
| Frontoparietal | Limbic | B3 | **141.84 (11.26)***** | | **-13.21 (3.59)***** | -0.44 (10.69) | **-24.98 (10.7)*** | **3.45 (1.48)*** | **4.29 (1.26)**** | - | -6.63  (16.31) | |  | | |
| Frontoparietal | Salience | B3 | **141.66 (11.27)***** | | **-13.48 (3.61)***** | -0.19 (10.68) | **-25.03 (10.69)*** | 2.75  (1.48) | **3.61 (1.20)**** | - | -6.74  (16.30) | |  | | |
| Frontoparietal | Somatomotor | B3 | **141.35 (11.28)***** | | **-13.60 (3.62)***** | -0.25 (10.68) | **-24.77 (10.69)*** | 3.81  (1.98) | **4.69 (1.66)*** | - | -6.59  (16.30) | |  | | |
| Frontoparietal | Visual | B3 | **141.38 (11.31)***** | | **-14.17 (3.67)***** | -0.31 (10.70) | **-25.23 (10.72)*** | 3.48  (1.85) | **4.78 (1.53)**** | - | -6.89  (16.34) | |  | | |
| Salience/ventral attention | Somatomotor | B3 | **140.09 (11.2)***** | | **-15.23 (3.64)***** | -0.73 (10.58) | **-25.24 (10.59)*** | **8.41 (2.58)**** | **8.67 (2.11)***** | - | -7.21  (16.18) | |  | | |

*Note*. Bold identifies significant independent variable following false discovery rate (FDR) correction from the Benjamini-Hochberg method (Benjamini & Hochberg, 1995). See equations for final models B1-4 in methods. All models include a random intercept and random age slope. Model including dorsal attention to frontoparietal functional connectivity (FC) also contained lower order interactions: FC x Group, -6.38 (3.73), *p* = .127; and Age x Group, -0.86 (7.36), *p* = .999. ADHD medication use represents dichotomous (yes/no) variable if any of methylphenidate, clonidine, atomoxetine or lisdexamfetamine were taken on an assessment day. * FDR-corrected *p* < .050, ** FDR-corrected *p* < .010, *** FDR-corrected *p* < .001, ^ *p* < .050 prior to FDR-correction only. Hyphen indicates variable was not involved in best fitting model.

**Models in motion-matched subsamples**

To ensure confidence in findings, two motion-matched subsamples were created following the matching approach of Satterthwaite et al. (2013). Briefly, for the first subsample a loop was created, and in each run a greedy matching algorithm identified and excluded the datapoint contributing most to the correlation between age and head motion (framewise displacement). The loop continued until the absolute age-motion correlation was less than 0.01. This process was conducted in ADHD and control groups separately to ensure age-motion correlations were negligible across groups and yielded an age-motion matched subsample of 148 participants (64 ADHD, 84 control, average datapoints per person = 2.1) where the overall correlation between age and motion was *r* = -0.006. For the second subsample, the same matching algorithm was applied only to participants with mean head motion less than 0.1mm, yielding a nearly motionless and motion-matched subsample of 90 participants (32 ADHD, 58 control, average datapoints per person = 1.5) where the correlation between age and motion was *r* = -0.001. Models were rerun in these two motion-matched subsamples with head motion included as a covariate in subsample 1 (see Tables S9-12). As medication use was small, and to provide further confidence in the findings, these subsamples included only participants without ADHD medication use. Results of interest were largely consistent when rerunning models in these two subsamples, except notably that no main effects of age survived FDR correction in functional connectivity models (see Tables S9 and S11).

Table S9. Linear Mixed Effects Models of Functional Connectivity Development (subsample 1, covarying for scanner motion)

| Connection of interest | |  | Variable [Regression Coefficient (standard error), FDR-corrected p-value] | | | | | | |
| --- | --- | --- | --- | --- | --- | --- | --- | --- | --- |
| Network 1 | Network 2 | Model form | (Intercept) | Age | Sex | Group | Age x Group | Head motion |  |
| Default Mode | Default Mode | A3 | **0.49 (0.04)***** | -0.01 (0.02) | 0.01 (0.04) | 0.01 (0.04) | 0.01 (0.02) | **1.04 (0.09)***** |  |
| Default Mode | Dorsal Attention | A3 | **0.30 (0.04)***** | -0.02 (0.02) | 0.06 (0.04) | 0.02 (0.03) | 0.03 (0.02) | **0.77 (0.08)***** |  |
| Default Mode | Frontoparietal | A3 | **0.30 (0.04)***** | -0.01 (0.02) | 0.06 (0.04) | 0.02 (0.04) | 0.01 (0.02) | **0.92 (0.07)***** |  |
| Default Mode | Limbic | A2 | **0.11 (0.04)*** | -0.01 (0.01) | 0.09 (0.04)* | 0.01 (0.04) | - | **0.94 (0.08)***** |  |
| Default Mode | Salience/Ventral Attention | A3 | **0.29 (0.04)***** | -0.01 (0.02) | 0.01 (0.04) | 0.01 (0.03) | 0.01 (0.02) | **0.91 (0.07)***** |  |
| Default Mode | Somatomotor | A3 | **0.36 (0.04)***** | -0.01 (0.02) | 0.04 (0.03) | 0.02 (0.03) | 0.00 (0.03) | **0.62 (0.07)***** |  |
| Default Mode | Visual | A1 | **0.51 (0.04)***** | - | 0.08 (0.03)* | 0.04 (0.03) | - | **0.63 (0.07)***** |  |
| Dorsal Attention | Dorsal Attention | A1 | **0.27 (0.05)***** | - | 0.05 (0.04) | 0.03 (0.04) | - | **0.96 (0.12)***** |  |
| Dorsal Attention | Frontoparietal | A2 | **0.42 (0.04)***** | -0.02 (0.01) | 0.00 (0.04) | 0.01 (0.04) | - | **0.80 (0.08)***** |  |
| Dorsal Attention | Limbic | A3 | **0.43 (0.05)***** | -0.02 (0.02) | 0.08 (0.04) | 0.01 (0.04) | 0.04 (0.03) | **1.13 (0.09)***** |  |
| Dorsal Attention | Salience/Ventral Attention | A3 | **0.58 (0.04)***** | 0.00 (0.02) | 0.03 (0.04) | 0.04 (0.04) | 0.01 (0.03) | **1.00 (0.09)***** |  |
| Dorsal Attention | Somatomotor | A2 | **0.40 (0.04)***** | -0.01 (0.01) | 0.06 (0.03) | 0.00 (0.03) | - | **0.51 (0.08)***** |  |
| Dorsal Attention | Visual | A1 | **0.36 (0.04)***** | - | 0.08 (0.04)^ | 0.05 (0.03) | - | **0.84 (0.09)***** |  |
| Frontoparietal | Frontoparietal | A2 | **0.13 (0.05)*** | 0.00 (0.01) | 0.05 (0.04) | 0.01 (0.04) | - | **1.09 (0.10)***** |  |
| Frontoparietal | Limbic | A3 | **0.21 (0.04)***** | -0.01 (0.02) | 0.04 (0.04) | 0.01 (0.04) | 0.00 (0.02) | **1.03 (0.08)***** |  |
| Frontoparietal | Salience/Ventral Attention | A3 | **0.41 (0.04)***** | -0.03 (0.02) | 0.04 (0.03) | 0.02 (0.03) | 0.03 (0.02) | **0.75 (0.08)***** |  |
| Frontoparietal | Somatomotor | A1 | **0.28 (0.04)***** | - | 0.04 (0.04) | 0.00 (0.04) | - | **0.80 (0.07)***** |  |
| Frontoparietal | Visual | A3 | **0.34 (0.04)***** | -0.02 (0.02) | 0.08 (0.03)^ | 0.04 (0.03) | 0.04 (0.02) | **0.80 (0.07)***** |  |
| Limbic | Limbic | A1 | **0.49 (0.05)***** | - | 0.03 (0.04) | 0.03 (0.04) | - | 0.20 (0.12) |  |
| Limbic | Salience/Ventral Attention | A3 | **0.34 (0.04)***** | -0.01 (0.02) | 0.02 (0.04) | 0.01 (0.04) | 0.01 (0.02) | **0.76 (0.08)***** |  |
| Limbic | Somatomotor | A2 | **0.30 (0.04)***** | -0.01 (0.01) | 0.04 (0.04) | 0.03 (0.04) | - | **0.77 (0.09)***** |  |
| Limbic | Visual | A3 | **0.41 (0.04)***** | -0.03 (0.02) | 0.08 (0.03)^ | 0.02 (0.03) | 0.04 (0.02) | **0.54 (0.08)***** |  |
| Salience/Ventral Attention | Salience/Ventral Attention | A1 | **0.57 (0.06)***** | - | 0.05 (0.05) | 0.00 (0.05) | - | **0.81 (0.13)***** |  |
| Salience/Ventral Attention | Somatomotor | A3 | **0.21 (0.05)***** | -0.04 (0.02) | 0.07 (0.04) | 0.01 (0.04) | 0.05 (0.03) | **0.81 (0.09)***** |  |
| Salience/Ventral Attention | Visual | A3 | **0.64 (0.04)***** | -0.03 (0.02) | **0.09 (0.03)*** | 0.03 (0.03) | 0.04 (0.02)^ | **0.58 (0.08)***** |  |
| Somatomotor | Somatomotor | A1 | **0.41 (0.05)***** | - | 0.07 (0.04) | 0.02 (0.04) | - | **0.36 (0.11)**** |  |
| Somatomotor | Visual | A1 | **0.39 (0.04)***** | - | 0.06 (0.04) | 0.02 (0.03) | - | **0.52 (0.08)***** |  |
| Visual | Visual | A1 | **0.98 (0.04)***** | - | 0.01 (0.04) | -0.03 (0.04) | - | 0.06 (0.10) |  |

*Note* Bold identifies significant independent variable following false discovery rate (FDR) correction from the Benjamini-Hochberg method (Benjamini & Hochberg, 1995). See equations for final models A1-3 in methods. All models include a random intercept; models with age variable include a random age slope. Following recommendations from Yan et al. (2013), head motion was measured using the framewise displacement approach of Jenkinson et al. (2002). * FDR-corrected *p* < .050, ** FDR-corrected *p* < .010, *** FDR-corrected *p* < .001, ^ *p* < .050 prior to FDR-correction only. Hyphen indicates variable was not involved in best fitting model.

| Table S10. Linear Mixed Effects Models of Sustained Attention Development (subsample 1, covarying for scanner motion) | | | | | | | | | | | | | | |  |
| --- | --- | --- | --- | --- | --- | --- | --- | --- | --- | --- | --- | --- | --- | --- | --- |
| Connection of interest | |  | | Variable [Regression Coefficient (standard error), FDR-corrected p-value] | | | | | | | | | |  |  |
| Network 1 | Network 2 | Model form | (Intercept) | | Age | Sex | Group | FC | FC x Age | FC x Age x Group | Head motion |  |  |  |  |
| Default Mode | Frontoparietal | B3 | **124.03 (12.04)***** | | **-14.40 (4.21)**** | -12.45 (11.28) | -22.30 (11.12)^ | 1.21  (1.25) | **3.80 (1.10)**** | - | **101.70 (7.97)***** | |  | | |
| Default Mode | Limbic | B3 | **124.47 (12.04)***** | | **-13.93 (4.19)**** | -13.00 (11.28) | -22.22 (11.11)^ | 2.02  (1.30) | **4.23 (1.16)***** | - | **100.82 (8.02)***** | |  | | |
| Default Mode | Salience/ventral attention | B3 | **123.87 (12.05)***** | | **-15.34 (4.23)***** | -12.57 (11.29) | -22.19 (11.12)^ | 0.96  (1.56) | **5.77 (1.35)***** | - | **103.13 (8.02)***** | |  | | |
| Dorsal Attention | Frontoparietal | B4 | **115.92 (12.41)***** | | **-18.10 (7.23)*** | -11.79 (11.27) | -9.55 (11.98) | **15.11 (3.09)***** | **14.09 (2.81)***** | **-10.12 (3.6)**** | **92.48 (9.25)***** | |  | | |
| Dorsal Attention | Limbic | B3 | **123.3 (12.25)***** | | **-16.46 (4.40)***** | -12.74 (11.28) | -22.55 (11.11)^ | **5.39 (2.43)*** | **5.47 (2.05)*** | - | **91.91 (11.53)***** | |  | | |
| Frontoparietal | Frontoparietal | B3 | **126.68 (12.14)***** | | **-15.01 (4.18)***** | -12.58 (11.14) | -23.12 (10.97)^ | 5.29 (2.53)^ | **6.60 (2.25)**** | - | **86.98 (13.47)***** | |  | | |
| Frontoparietal | Limbic | B3 | **124.43 (12.09)***** | | **-14.97 (4.20)***** | -12.73 (11.28) | -22.31 (11.11)^ | 1.86  (1.56) | **5.65 (1.38)***** | - | **100.01 (9.31)***** | |  | | |
| Frontoparietal | Salience | B3 | **123.52 (12.08)***** | | **-15.22 (4.24)***** | -12.56 (11.25) | -22.45 (11.08)^ | 2.83  (1.57) | **4.69 (1.39)**** | - | **100.03 (9.22)***** | |  | | |
| Frontoparietal | Somatomotor | B3 | **123.54 (12.04)***** | | **-15.68 (4.25)***** | -12.56 (11.22) | -22.04 (11.06)^ | 3.16  (2.18) | **6.69 (1.91)**** | - | **99.66 (9.35)***** | |  | | |
| Frontoparietal | Visual | B3 | **124.15 (12.11)***** | | **-16.20 (4.32)***** | -12.53 (11.29) | -22.54 (11.12)^ | 1.85  (2.08) | **6.19 (1.82)**** | - | **99.93 (9.33)***** | |  | | |
| Salience/ventral attention | Somatomotor | B3 | **123.81 (12.08)***** | | **-17.49 (4.29)***** | -12.66 (11.16) | -22.60 (11.00)^ | **7.01**  **(2.80)*** | **11.24 (2.41)***** | - | **94.07 (11.41)***** | |  | | |

*Note*. Bold identifies significant independent variable following false discovery rate (FDR) correction from the Benjamini-Hochberg method (Benjamini & Hochberg, 1995). See equations for final models B1-4 in methods. All models include a random intercept and random age slope. Model including dorsal attention to frontoparietal functional connectivity (FC) also contained lower order interactions: FC x Group, -17.31 (4.04), *p* < .001; and Age x Group, 1.62 (9.02), *p* = .858. Following recommendations from Yan et al. (2013), head motion was measured using the framewise displacement approach of Jenkinson et al. (2002). * FDR-corrected *p* < .050, ** FDR-corrected *p* < .010, *** FDR-corrected *p* < .001, ^ *p* < .050 prior to FDR-correction only. Hyphen indicates variable was not involved in best fitting model.

Table S11. Linear Mixed Effects Models of Functional Connectivity Development (subsample 2)

| Connection of interest | | |  | | Variable [Regression Coefficient (standard error), FDR-corrected p-value] | | | | | | |  |
| --- | --- | --- | --- | --- | --- | --- | --- | --- | --- | --- | --- | --- |
| Network 1 | Network 2 | Model form | | (Intercept) | | Age | Sex | Group | Age x Group |  |  | |
| Default Mode | Default Mode | A3 | | **0.54 (0.05)***** | | -0.02 (0.03) | 0.02 (0.05) | 0.04 (0.05) | 0.03 (0.03) |  |  | |
| Default Mode | Dorsal Attention | A3 | | **0.36 (0.05)***** | | -0.05 (0.03) | 0.05 (0.05) | 0.03 (0.05) | 0.06 (0.04) |  |  | |
| Default Mode | Frontoparietal | A3 | | **0.37 (0.05)***** | | -0.04 (0.03) | 0.05 (0.05) | 0.04 (0.05) | 0.05 (0.03) |  |  | |
| Default Mode | Limbic | A2 | | **0.16 (0.05)**** | | -0.01 (0.02) | 0.10 (0.05)^ | 0.04 (0.05) | - |  |  | |
| Default Mode | Salience/Ventral Attention | A3 | | **0.32 (0.05)***** | | -0.03 (0.03) | 0.04 (0.04) | 0.05 (0.05) | 0.03 (0.04) |  |  | |
| Default Mode | Somatomotor | A3 | | **0.39 (0.05)***** | | -0.03 (0.03) | 0.04 (0.04) | 0.05 (0.04) | 0.04 (0.03) |  |  | |
| Default Mode | Visual | A1 | | **0.54 (0.04)***** | | - | 0.07 (0.04) | 0.08 (0.04) | - |  |  | |
| Dorsal Attention | Dorsal Attention | A1 | | **0.32 (0.07)***** | | - | 0.02 (0.06) | 0.09 (0.06) | - |  |  | |
| Dorsal Attention | Frontoparietal | A2 | | **0.47 (0.05)***** | | -0.02 (0.02) | 0.00 (0.05) | 0.06 (0.05) | - |  |  | |
| Dorsal Attention | Limbic | A3 | | **0.49 (0.06)***** | | -0.04 (0.03) | 0.13 (0.06)^ | 0.03 (0.06) | 0.08 (0.03)^ |  |  | |
| Dorsal Attention | Salience/Ventral Attention | A3 | | **0.66 (0.05)***** | | -0.03 (0.04) | 0.05 (0.05) | 0.06 (0.05) | 0.02 (0.04) |  |  | |
| Dorsal Attention | Somatomotor | A2 | | **0.43 (0.05)***** | | -0.01 (0.02) | 0.05 (0.04) | 0.03 (0.04) | - |  |  | |
| Dorsal Attention | Visual | A1 | | **0.42 (0.05)***** | | - | 0.06 (0.04) | 0.09 (0.05) | - |  |  | |
| Frontoparietal | Frontoparietal | A2 | | **0.19 (0.05)**** | | 0.00 (0.02) | 0.05 (0.05) | 0.04 (0.05) | - |  |  | |
| Frontoparietal | Limbic | A3 | | **0.28 (0.05)***** | | -0.03 (0.03) | 0.06 (0.05) | 0.04 (0.05) | 0.02 (0.04) |  |  | |
| Frontoparietal | Salience/Ventral Attention | A3 | | **0.44 (0.05)***** | | -0.07 (0.03)^ | 0.08 (0.04) | 0.04 (0.05) | **0.09 (0.03)*** |  |  | |
| Frontoparietal | Somatomotor | A1 | | **0.31 (0.05)***** | | - | 0.08 (0.05) | 0.03 (0.05) | - |  |  | |
| Frontoparietal | Visual | A3 | | **0.39 (0.05)***** | | -0.03 (0.02) | 0.07 (0.04) | 0.08 (0.05) | 0.05 (0.03) |  |  | |
| Limbic | Limbic | A1 | | **0.37 (0.06)***** | | - | 0.07 (0.06) | 0.11 (0.06) | - |  |  | |
| Limbic | Salience/Ventral Attention | A3 | | **0.39 (0.05)***** | | -0.05 (0.03) | 0.04 (0.05) | -0.01 (0.05) | 0.06 (0.04) |  |  | |
| Limbic | Somatomotor | A2 | | **0.35 (0.05)***** | | -0.02 (0.02) | 0.05 (0.05) | 0.03 (0.05) | - |  |  | |
| Limbic | Visual | A3 | | **0.45 (0.04)***** | | -0.04 (0.02) | 0.07 (0.04) | 0.04 (0.04) | 0.06 (0.03)^ |  |  | |
| Salience/Ventral Attention | Salience/Ventral Attention | A1 | | **0.58 (0.07)***** | | - | 0.13 (0.07) | -0.01 (0.07) | - |  |  | |
| Salience/Ventral Attention | Somatomotor | A3 | | **0.21 (0.05)***** | | -0.04 (0.03) | 0.09 (0.05) | 0.09 (0.05) | 0.08 (0.04)^ |  |  | |
| Salience/Ventral Attention | Visual | A3 | | **0.67 (0.05)***** | | -0.05 (0.03)^ | 0.09 (0.04)^ | 0.06 (0.05) | **0.10 (0.03)**** |  |  | |
| Somatomotor | Somatomotor | A1 | | **0.33 (0.06)***** | | - | 0.11 (0.05) | 0.1 (0.06) | - |  |  | |
| Somatomotor | Visual | A1 | | **0.40 (0.05)***** | | - | 0.07 (0.04) | 0.05 (0.05) | - |  |  | |
| Visual | Visual | A1 | | **0.99 (0.05)***** | | - | -0.02 (0.04) | -0.02 (0.05) | - |  |  | |

*Note* Bold identifies significant independent variable following false discovery rate (FDR) correction from the Benjamini-Hochberg method (Benjamini & Hochberg, 1995). See equations for final models A1-3 in methods. All models include a random intercept; models with age variable include a random age slope. * FDR-corrected *p* < .050, ** FDR-corrected *p* < .010, *** FDR-corrected *p* < .001, ^ *p* < .050 prior to FDR-correction only. Hyphen indicates variable was not involved in best fitting model.

| Table S12. Linear Mixed Effects Models of Sustained Attention Development (subsample 2) | | | | | | | | | | | | | |  |
| --- | --- | --- | --- | --- | --- | --- | --- | --- | --- | --- | --- | --- | --- | --- |
| Connection of interest | |  | | Variable [Regression Coefficient (standard error), FDR-corrected p-value] | | | | | | | | |  |  |
| Network 1 | Network 2 | Model form | (Intercept) | | Age | Sex | Group | FC | FC x Age | FC x Age x Group |  |  |  |  |
| Default Mode | Frontoparietal | B3 | **141.73 (14.79)***** | | -10.85 (7.46) | -16.03 (13.54) | -17.10 (14.17) | 0.27 (0.65) | **1.94 (0.53)**** | - |  | |  | |
| Default Mode | Limbic | B3 | **141.84 (14.79)***** | | -10.50 (7.46) | -16.11 (13.55) | -16.97 (14.17) | 0.01 (0.62) | **1.68 (0.54)**** | - |  | |  | |
| Default Mode | Salience/ventral attention | B3 | **141.52 (14.77)***** | | -11.18 (7.46) | -15.97 (13.53) | -17.08 (14.16) | 0.82 (0.80) | **3.04 (0.66)***** | - |  | |  | |
| Dorsal Attention | Frontoparietal | B4 | **141.61 (14.96)***** | | -10.88 (13.46) | -15.88 (13.56) | -17.53 (14.75) | -0.02 (1.76) | 1.45  (1.59) | **6.77 (1.96)**** |  | |  | |
| Dorsal Attention | Limbic | B3 | **141.47 (14.8)***** | | -11.35 (7.48) | -16.07 (13.54) | -16.95 (14.17) | 0.48 (1.32) | 2.25 (1.08)^ | - |  | |  | |
| Frontoparietal | Frontoparietal | B3 | **141.89 (14.8)***** | | -11.11 (7.46) | -15.79 (13.56) | -17.04 (14.18) | -0.96 (1.41) | **4.03 (1.15)**** | - |  | |  | |
| Frontoparietal | Limbic | B3 | **142.2 (14.8)***** | | -10.94 (7.47) | -16.05 (13.55) | -16.96 (14.18) | -1.27 (0.76) | **2.53 (0.64)***** | - |  | |  | |
| Frontoparietal | Salience | B3 | **141.57 (14.77)***** | | -11.08 (7.46) | -16.08 (13.52) | -17.08 (14.15) | 0.54 (0.81) | **2.04 (0.66)**** | - |  | |  | |
| Frontoparietal | Somatomotor | B3 | **141.54 (14.77)***** | | -11.54 (7.46) | -16.02 (13.53) | -16.95 (14.15) | 0.54 (1.27) | **3.79 (1.01)***** | - |  | |  | |
| Frontoparietal | Visual | B3 | **141.1 (14.78)***** | | -11.32 (7.47) | -16.14 (13.54) | -17.22 (14.16) | 1.75 (1.11) | **2.63 (0.93)*** | - |  | |  | |
| Salience/ventral attention | Somatomotor | B3 | **141.81 (14.76)***** | | -11.89 (7.48) | -16.08 (13.51) | -17.31 (14.14) | 0.44 (1.56) | **5.65 (1.28)***** | - |  | |  | |

*Note*. Bold identifies significant independent variable following false discovery rate (FDR) correction from the Benjamini-Hochberg method (Benjamini & Hochberg, 1995). See equations for final models B1-4 in methods. All models include a random intercept and random age slope. Model including dorsal attention to frontoparietal functional connectivity (FC) also contained lower order interactions: FC x Group, 1.44 (2.29), *p* = .589; and Age x Group, -3.51 (16.15), *p* = .852. * FDR-corrected *p* < .050, ** FDR-corrected *p* < .010, *** FDR-corrected *p* < .001, ^ *p* < .050 prior to FDR-correction only. Hyphen indicates variable was not involved in best fitting model.

Table S13. Pearson correlation of framewise displacement and network connectivity

| Connection of interest | |  |
| --- | --- | --- |
| Network 1 | Network 2 | Pearson correlation |
| Default Mode | Default Mode | .50, *p* < .001 |
| Default Mode | Dorsal Attention | .52, *p* < .001 |
| Default Mode | Frontoparietal | .58, *p* < .001 |
| Default Mode | Limbic | .16, *p* = .066 |
| Default Mode | Salience/Ventral Attention | .44, *p* < .001 |
| Default Mode | Somatomotor | .40, *p* < .001 |
| Default Mode | Visual | .48, *p* < .001 |
| Dorsal Attention | Dorsal Attention | .34, *p* < .001 |
| Dorsal Attention | Frontoparietal | .38, *p* < .001 |
| Dorsal Attention | Limbic | .36, *p* < .001 |
| Dorsal Attention | Salience/Ventral Attention | .39, *p* < .001 |
| Dorsal Attention | Somatomotor | .45, *p* < .001 |
| Dorsal Attention | Visual | .29, *p* < .001 |
| Frontoparietal | Frontoparietal | .43, *p* < .001 |
| Frontoparietal | Limbic | .31, *p* < .001 |
| Frontoparietal | Salience/Ventral Attention | .40, *p* < .001 |
| Frontoparietal | Somatomotor | .43, *p* < .001 |
| Frontoparietal | Visual | .37, *p* < .001 |
| Limbic | Limbic | .21, *p* = .029 |
| Limbic | Salience/Ventral Attention | .22, *p* = .009 |
| Limbic | Somatomotor | .20, *p* = .020 |
| Limbic | Visual | .33, *p* < .001 |
| Salience/Ventral Attention | Salience/Ventral Attention | .20, *p* = .043 |
| Salience/Ventral Attention | Somatomotor | .34, *p* < .001 |
| Salience/Ventral Attention | Visual | .35, *p* < .001 |
| Somatomotor | Somatomotor | .11, *p* = .273 |
| Somatomotor | Visual | .43, *p* < .001 |
| Visual | Visual | .06, *p* = .555 |

References – Supplementary Materials

Abraham, A., Pedregosa, F., Eickenberg, M., Gervais, P., Mueller, A., Kossaifi, J., . . . Varoquaux, G. (2014). Machine learning for neuroimaging with scikit-learn. *Frontiers in Neuroinformatics, 8*, 14.

Australian Bureau of Statistics. (2013). Census of population and housing: Socio-economic indexes for areas (SEIFA), Australia, 2011. *cat. no. 2033.0. 55.001*.

Avants, B. B., Epstein, C. L., Grossman, M., & Gee, J. C. (2008). Symmetric diffeomorphic image registration with cross-correlation: evaluating automated labeling of elderly and neurodegenerative brain. *Medical Image Analysis, 12*(1), 26-41.

Behzadi, Y., Restom, K., Liau, J., & Liu, T. T. (2007). A component based noise correction method (CompCor) for BOLD and perfusion based fMRI. *Neuroimage, 37*(1), 90-101.

Benjamini, Y., & Hochberg, Y. (1995). Controlling the false discovery rate: a practical and powerful approach to multiple testing. *Journal of the Royal statistical society: series B (Methodological), 57*(1), 289-300.

Cox, R. W., & Hyde, J. S. (1997). Software tools for analysis and visualization of fMRI data. *NMR in Biomedicine: An International Journal Devoted to the Development and Application of Magnetic Resonance In Vivo, 10*(4‐5), 171-178.

Evans, A. C., Janke, A. L., Collins, D. L., & Baillet, S. (2012). Brain templates and atlases. *Neuroimage, 62*(2), 911-922.

Fonov, V. S., Evans, A. C., McKinstry, R. C., Almli, C., & Collins, D. (2009). Unbiased nonlinear average age-appropriate brain templates from birth to adulthood. *Neuroimage*(47), S102.

Greve, D. N., & Fischl, B. (2009). Accurate and robust brain image alignment using boundary-based registration. *Neuroimage, 48*(1), 63-72.

Jenkinson, M., Bannister, P., Brady, M., & Smith, S. (2002). Improved optimization for the robust and accurate linear registration and motion correction of brain images. *Neuroimage, 17*(2), 825-841.

Jenkinson, M., & Smith, S. (2001). A global optimisation method for robust affine registration of brain images. *Medical Image Analysis, 5*(2), 143-156.

Johnson, K. A., Robertson, I. H., Kelly, S. P., Silk, T. J., Barry, E., Dáibhis, A., . . . Gallagher, L. (2007). Dissociation in performance of children with ADHD and high-functioning autism on a task of sustained attention. *Neuropsychologia, 45*(10), 2234-2245.

Lanczos, C. (1964). Evaluation of noisy data. *Journal of the Society for Industrial and Applied Mathematics, Series B: Numerical Analysis, 1*(1), 76-85.

Power, J. D., Mitra, A., Laumann, T. O., Snyder, A. Z., Schlaggar, B. L., & Petersen, S. E. (2014). Methods to detect, characterize, and remove motion artifact in resting state fMRI. *Neuroimage, 84*, 320-341.

Pruim, R. H., Mennes, M., van Rooij, D., Llera, A., Buitelaar, J. K., & Beckmann, C. F. (2015). ICA-AROMA: A robust ICA-based strategy for removing motion artifacts from fMRI data. *Neuroimage, 112*, 267-277. doi:10.1016/j.neuroimage.2015.02.064

Satterthwaite, T. D., Wolf, D. H., Ruparel, K., Erus, G., Elliott, M. A., Eickhoff, S. B., . . . Smith, A. (2013). Heterogeneous impact of motion on fundamental patterns of developmental changes in functional connectivity during youth. *Neuroimage*, 83, 45-57.

Shaffer, D., Fisher, P., Lucas, C. P., Dulcan, M. K., & Schwab-Stone, M. E. (2000). NIMH Diagnostic Interview Schedule for Children Version IV (NIMH DISC-IV): description, differences from previous versions, and reliability of some common diagnoses. *Journal of the American Academy of Child and Adolescent Psychiatry, 39*(1), 28-38.

Thomson, P., Johnson, K. A., Malpas, C. B., Efron, D., Sciberras, E., & Silk, T. J. (2021). Head Motion During MRI Predicted by out-of-Scanner Sustained Attention Performance in Attention-Deficit/Hyperactivity Disorder. *Journal of attention disorders, 25*(10), 1429-1440.

Tustison, N. J., Avants, B. B., Cook, P. A., Zheng, Y., Egan, A., Yushkevich, P. A., & Gee, J. C. (2010). N4ITK: improved N3 bias correction. *IEEE Transactions on Medical Imaging, 29*(6), 1310-1320.

Vijayakumar, N., Youssef, G. J., Allen, N. B., Anderson, V., Efron, D., Hazell, P., . . . Seal, M. L. (2021). A longitudinal analysis of puberty-related cortical development. *Neuroimage, 228*, 117684.

Wechsler, D. (1999). Manual for the Wechsler abbreviated intelligence scale (WASI). *San Antonio, TX: The Psychological Corporation*.

Yan, C.-G., Cheung, B., Kelly, C., Colcombe, S., Craddock, R. C., Di Martino, A., . . . Milham, M. P. (2013). A comprehensive assessment of regional variation in the impact of head micromovements on functional connectomics. *Neuroimage, 76*, 183-201.

Zhang, Y., Brady, M., & Smith, S. (2001). Segmentation of brain MR images through a hidden Markov random field model and the expectation-maximization algorithm. *IEEE Transactions on Medical Imaging, 20*(1), 45-57.
